# Personalized Geriatric Pharmacotherapy: Pharmacogenomics, Polypharmacy, and Age-Related Pharmacokinetic and Pharmacodynamic Changes

**DOI:** 10.3390/ijms27219948

**Published:** 2026-11-06

**Authors:** Anastasiia A. Buianova, Iaroslav V. Mironenko, Mikhail S. Arbatskiy, Alexey V. Churov

**Affiliations:** Russian Clinical Research Center of Gerontology, Pirogov Russian National Research Medical University of the Ministry of Healthcare of the Russian Federation, 129226 Moscow, Russia; jarosmironen@gmail.com (I.V.M.); algenubi81@mail.ru (M.S.A.)

**Keywords:** aging, pharmacokinetics, pharmacodynamics, polypharmacy, pharmacogenomics, geriatric pharmacotherapy, adverse drug reactions

## Abstract

Aging induces profound physiological changes that alter drug disposition and response, narrowing therapeutic windows and increasing susceptibility to adverse drug reactions (ADRs). This review synthesizes current evidence on age-related pharmacokinetic and pharmacodynamic changes, with a focus on pharmacogenomic determinants of drug response in older adults. Progressive decline in renal function and hepatic metabolic capacity and alterations in body composition collectively necessitate age-appropriate dose adjustments. Polypharmacy, prevalent in community-dwelling and hospitalized older adults, compounds these challenges through drug–drug and drug–drug–gene interactions. Pharmacogenomic testing—particularly for *CYP2D6*, *CYP2C19*, *CYP2C9*, and *SLCO1B1*—offers a promising strategy with which to personalize therapy, while deprescribing frameworks provide practical approaches to mitigating medication-related risks. This review provides a comprehensive framework for understanding the mechanistic basis of altered drug response in older adults and proposes actionable strategies for optimizing pharmacotherapy in this vulnerable population. Throughout, pharmacogenomic claims are graded across four levels: biological plausibility, measured pharmacokinetic effect, pharmacogenetic actionability, and demonstrated clinical utility. Furthermore, selected gene–drug examples are explicitly identified as either geriatric-specific evidence or extrapolations from general adult pharmacogenomics, clarifying which findings are truly modified by the aging process.

## 1. Introduction

Geriatric patients, defined as those aged ≥65 years, are among the highest consumers of medications worldwide [1]. The complexity of pharmacotherapy in this population is increased due to age-related physiological changes, multiple coexisting diseases, and the concurrent use of several medications, prescribers, and pharmacies. The most significant aspect of aging is the gradual reduction in functional units over time—the smallest structures able to carry out the specific physiological roles of their respective organs, such as nephrons in the kidneys, alveoli in the lungs, or neurons in the brain [2,3]. Another characteristic is the disruption of regulatory processes that ensure functional integration between cells and organs, reducing the ability to maintain homeostasis under physiological stress [3,4].

Older adults consume a disproportionate share of medications. For clarity, the following operational terminology is used throughout this review: polypharmacy denotes the concurrent use of five or more medications; excessive polypharmacy and hyperpolypharmacy are treated as synonyms and denote the concurrent use of ten or more medications. Polypharmacy affects 30–40% of community-dwelling individuals over 65 years, and the rate of hyperpolypharmacy (ten or more medications) reaches 39–45% in geriatric and psychiatric inpatient settings [1,5,6]. In the ASPREE cohort, 98.8% of participants carried at least one actionable pharmacogenetic genotype, with polypharmacy (defined as ≥5 medications) affecting 83.9% and CYP450 inhibitor/inducer use in 68.2%. However, only 27.5% received a medication for which a deviation from standard prescribing would be recommended based on their genotype [7].

**Figure 1:**
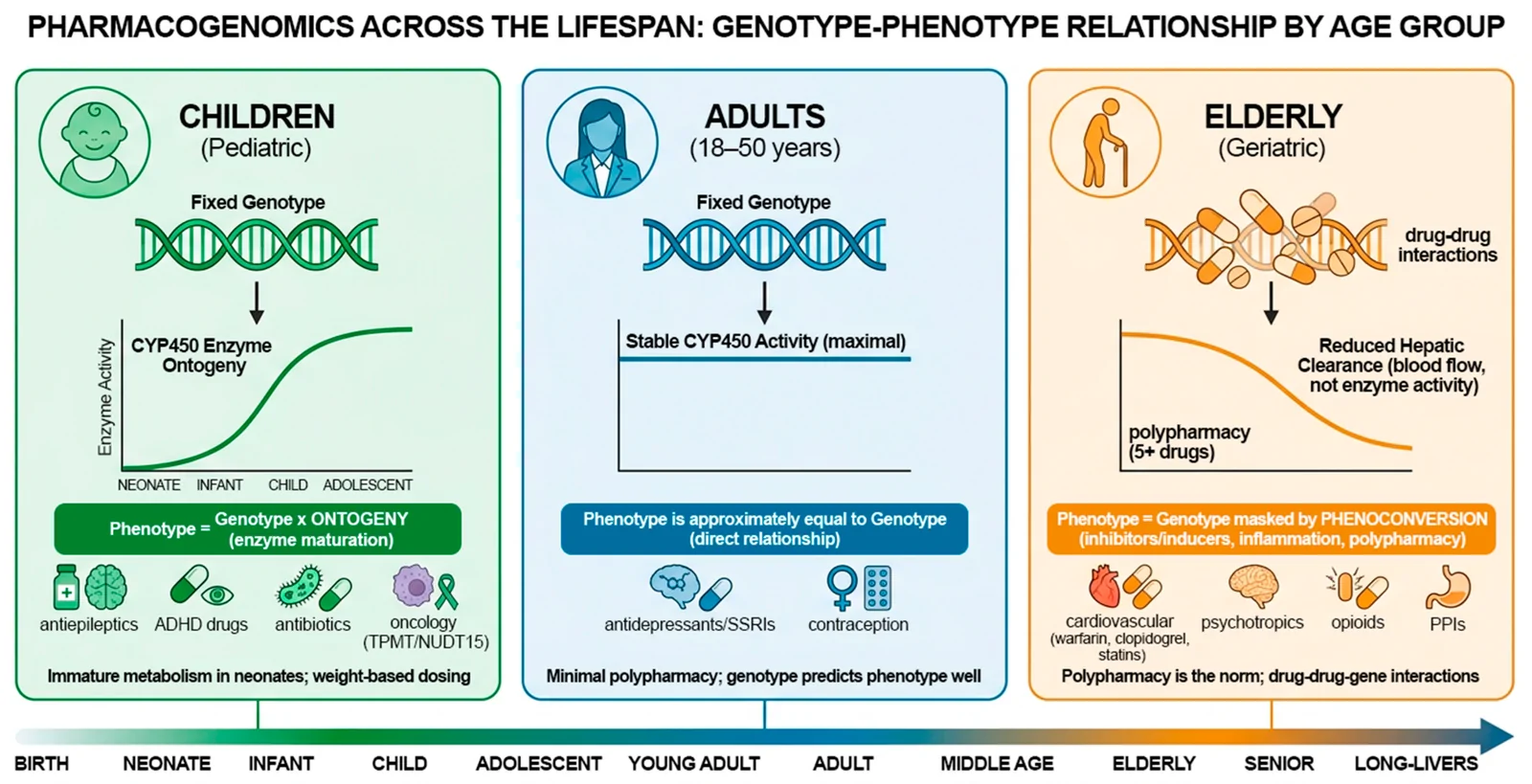
The pharmacogene panel remains identical across the lifespan, but the interpretation of genotype–phenotype relationships requires age-specific considerations. Ontogeny (enzyme maturation) influences response in children, while phenoconversion due to factors like polypharmacy, drug–drug interactions, and physiological changes masks the genotype in the elderly.

The physiological changes accompanying aging profoundly affect both pharmacokinetics (what the body does to the drug) and pharmacodynamics (what the drug does to the body) (Figure 1) [8]. These alterations, combined with the high prevalence of polypharmacy, create a substantial risk of adverse drug reactions (ADRs) [9]. Importantly, many of these ADRs are preventable through appropriate dose adjustment, drug selection, and pharmacogenomics-guided prescribing [10,11,12].

This review integrates evidence on age-related pharmacokinetic and pharmacodynamic changes, polypharmacy, and pharmacogenomic variability into a single framework for personalized geriatric pharmacotherapy.

Two scope decisions follow from this aim and are applied consistently throughout. First, the review is organised around pharmacogenomics rather than around geriatric pharmacotherapy in general: the clinical sections (Section 6, Section 7, Section 8 and Section 9) focus on how aging modifies a genotype–phenotype or genotype–exposure relationship, rather than on general therapeutic recommendations. Second, we separate three claims that are easily conflated—that a pharmacogenetic association exists, that it is actionable under a published prescribing guideline, and that it matters more in older adults than in younger adults. Only the third is a geriatric claim. Most pharmacogenetic associations are themselves not age-specific because allele frequencies do not change with age; what is specific to older adults is the context in which those associations operate—a much higher prevalence of exposure to actionable drugs, a markedly higher probability of phenoconversion through co-medication, reduced hepatic and renal reserve that shifts the dose–exposure relationship for an unchanged genotype, and a narrower margin between a tolerated and a harmful exposure. Where age-stratified comparisons with younger cohorts are available, we report them; where they are not, the example is labelled as an extrapolation from general adult pharmacogenomics rather than presented as geriatric evidence.

## 2. Aging and Alterations in Drug Response

### 2.1. Pharmacokinetic Changes

Pharmacokinetics involves the movement of drugs and their metabolites through the body, covering absorption, distribution, metabolism, and excretion (ADME) [3]. Aging induces significant structural and functional changes across all organ systems, affecting each of these processes.

#### 2.1.1. Drug Absorption

Age-related changes in gastrointestinal physiology have modest but clinically significant effects on drug absorption [2,13].

**Stomach.** Gastric acid secretion declines with age (hypochlorhydria), increasing gastric pH [2]. Age-related increases in gastric pH primarily affect the dissolution of weakly basic drugs such as ketoconazole and itraconazole, potentially reducing their systemic absorption. In contrast, increased gastric pH may theoretically reduce gastric membrane permeability for weak acids by increasing their degree of ionization; however, this effect is generally of limited clinical importance because absorption of most drugs occurs predominantly in the small intestine rather than the stomach. Gastric emptying also slows with advancing age, which can paradoxically increase the absorption of some drugs while decreasing that of others. Delayed gastric emptying particularly affects drugs that are unstable in acidic environments, as it postpones their arrival at the preferred absorption site [3]. A notable exception is calcium carbonate, which requires an acidic environment for optimal absorption; delayed gastric motility can instead benefit this drug by prolonging its presence in the stomach [3,14].

**Small intestine*****.*** Active transport processes, including those for calcium, iron, thiamine, and folic acid, are substantially impaired in the aging gastrointestinal tract [2,13]. Furthermore, reduced splanchnic blood flow and decreased intestinal mucosal surface area [3,15,16], as well as a significant decline in pancreatic enzyme secretion [17], contribute to altered intestinal absorption.

**Other extravascular routes*****.*** Beyond the gastrointestinal tract, the rate of absorption from subcutaneous and intramuscular sites may be slowed by reduced tissue blood perfusion in older adults, and intramuscular administration is associated with erratic and less predictable absorption. With respect to the pulmonary route, age-related declines in chest-wall compliance, ventilation–perfusion matching, and alveolar surface area reduce the efficiency of pulmonary gas exchange [18] and may, in principle, limit the pulmonary uptake of inhaled agents.

#### 2.1.2. Drug Distribution

The distribution of medications throughout the body in geriatric patients differs significantly from that in younger patients, which can lead to potential overdose risks. Geriatric patients typically experience a 20–40% increase in body fat, alongside a 10–15% decrease in lean body mass and total body water [3,6,13]. This altered composition affects the distribution volume of hydrophilic and lipophilic drugs.

Lipophilic drugs such as chlordiazepoxide, morphine, amiodarone, and diazepam tend to have a larger volume of distribution in geriatric patients, creating a larger reservoir within the body [3,6]. Because the elimination half-life is proportional to the volume of distribution at a given clearance (*t*1/2 = 0.693 × Vd/CL), lipophilic drugs with a larger Vd exhibit longer half-lives, resulting in extended durations of action and an increased risk of residual effects [3]. Conversely, hydrophilic drugs such as digoxin, lithium, and theophylline have a reduced distribution volume, leading to higher plasma concentrations and necessitating smaller doses to achieve therapeutic levels [3,19,20]. For water-soluble drugs, the reduced volume of distribution is often balanced by decreased renal clearance, with little net effect on the elimination half-life [3,19].

Plasma protein binding is altered in older adults. Acidic drugs such as phenytoin, warfarin, and acetylsalicylic acid primarily bind to albumin, while basic drugs like lidocaine and propranolol bind to α_1_-acid glycoprotein [3,21,22]. While concentrations of these proteins generally remain stable with age, albumin levels can decrease due to conditions like malnutrition or acute illness, whereas α_1_-acid glycoprotein levels may increase during acute kidney injury [22,23]. The unbound (free) concentration of a drug is crucial for its pharmacological effect and poses a higher risk of toxicity, particularly with highly protein-bound drugs such as phenytoin and warfarin [3]. In practice, polypharmacy is a more clinically important driver of altered free-drug exposure in older adults than the age-related change in albumin concentration itself: displacement interactions, in which one highly protein-bound drug competes for binding sites with another (e.g., valproate displacing phenytoin), combined with age-related reductions in the clearance of the displaced drug, can produce a more pronounced and clinically consequential rise in free-drug concentration than the modest, largely illness-driven decline in albumin [3,24].

An apparent paradox in the transporter literature deserves mention. In the intestine, targeted proteomics has identified increased abundance of several efflux transporters (BCRP, MRP1, MRP3, MRP4) with age [25], whereas at the blood–brain barrier, P-glycoprotein (P-gp) function appears to decrease with age, a change that may contribute to increased central nervous system drug exposure and is consistent with the heightened sensitivity to CNS depressants, including benzodiazepines, observed in older adults [26,27]. We propose three non-exclusive hypotheses for this divergence. First, the intestine is chronically exposed to xenobiotics and may upregulate efflux transporters as a compensatory barrier response to the cumulative lifetime exposure, whereas the blood–brain barrier lacks this continuous stimulus. Second, the two tissues differ in their cellular turnover and regulatory environment: enterocytes are renewed every 3–5 days, and their transporter expression is strongly influenced by luminal factors (diet, microbiota, drugs), whereas brain capillary endothelial cells are long-lived and their transporter expression is regulated by astrocyte-derived signals that change with age. Third, some of the observed changes may reflect methodological differences: intestinal transporter abundance was measured by targeted proteomics of duodenal biopsies, whereas blood–brain barrier P-gp function has been inferred mainly from positron emission tomography and cerebrospinal fluid studies, and the two methods are not directly comparable. Clinically, the divergence means that an age-related increase in intestinal efflux would be expected to reduce oral bioavailability of P-gp substrates (e.g., dabigatran etexilate), whereas a decrease in blood–brain barrier efflux would be expected to increase central nervous system exposure to the same substrates (e.g., loperamide, some opioids, some antipsychotics)—opposite directions of effect for the same transporter at two different sites, which is not captured by a single *ABCB1* genotype.

#### 2.1.3. Drug Metabolism

Hepatic drug metabolism undergoes substantial age-related decline, with reductions in both liver mass and overall in vivo metabolic capacity [3,6,13,28,29]. Total liver volume decreases by 20–30% in older adults, with more pronounced reductions in women. Hepatic blood flow declines by 35–50%, further reducing the clearance of drugs with high hepatic extraction ratios [3,28].

The systemic metabolic capacity of cytochrome P450 enzymes declines with age, though the degree of decline varies significantly by isoform [7,13,28]. Corton et al. [28] examined global gene expression in the livers of young (21–45 years) and old (69+ years) individuals, identifying age- and sex-dependent changes in xenobiotic metabolism genes. Multitissue analysis of ADME gene expression by McCoy [29] revealed distinct clustering patterns based on organ system identity, with both sex and age effects across multiple human organ systems.

To provide a systematic overview of the available evidence for each major enzyme and transporter, we compiled Table 1, which summarises reported changes, types of evidence, conflicting data, and interpretation caveats. Pathways for which the evidence indicates little or no age-related change are included, because a documented absence of an age effect is as useful for interpretation as a documented decline: it identifies the drugs for which a dose adjustment on account of age alone is not supported and for which genotype therefore remains the dominant source of between-patient variability. Pitavastatin (among the statins), the glucuronidated phase II substrates, and CYP2D6 are the three such cases in Table 1. For *CYP2D6*, in vivo probe studies indicate that its intrinsic activity is largely preserved with aging, and the modest fall in clearance predicted by PBPK simulation (31% for desipramine) reflects reduced liver mass and blood flow in the model rather than a loss of enzyme activity [30,31].

**Table 1:** Age-related changes in drug-metabolising enzymes and transporters.

Enzyme/Transporter	Reported Change with Aging	Type of Evidence	Conflicting or Limiting Evidence	Consequence for Interpretation
CYP3A	Midazolam has an AUC geometric mean ratio of 2.30 (95% CI 1.70–3.09) in healthy older adults vs. healthy young adults, and atorvastatin has an AUC ratio of 2.14 (1.52–3.02), both with prolonged half-lives [32]. In older adults with CKD the ratios were 2.90 and 4.15, respectively [32]. Modelled midazolam clearance falls 27% between ages 20–25 and 65–70 [30]	In vivo microdose probe cocktail (*n* = 20 young/16 older/17 older with CKD) [32]; PBPK simulation [30]	Small sample sizes; the study population was Thai, and CYP3A5*3 genotype was determined, but the cohort was not powered for genotype stratification [32]	The largest measured effect on AUC among the pathways listed. Applies to genotypically normal patients, so it operates on top of, not instead of, genotype-based dose adjustment
CYP2C19	Modelled S-mephenytoin clearance falls 39% between ages 20–25 and 65–70 [30]	PBPK simulation, not a clinical measurement [30]	S-mephenytoin was the one probe of five whose simulated clearance fell outside 3.5-fold of clinical study values [30]; direct in vivo probe data on older adults are sparse [31]	Combined with the high prevalence of actionable *CYP2C19* genotypes (59.6% [7]), the practical driver of exposure remains genotype plus co-medication
CYP2C9	Modelled S-warfarin clearance falls 36% between ages 20–25 and 65–70 [30]	PBPK simulation [30]	Direct in vivo age-stratified probe data limited [31]	Relevant to warfarin and NSAID exposure, but genotype remains the larger source of between-patient variability
CYP1A2	Modelled caffeine clearance falls 33% between ages 20–25 and 65–70 [30]	PBPK simulation [30]	Inducibility by smoking is a separate and larger source of variability	No actionable pharmacogenetic guidelines; relevant mainly as a background covariate for clozapine and theophylline
CYP2D6	Modelled desipramine clearance falls 31% between ages 20–25 and 65–70 [30]. In vivo probe studies indicate that intrinsic CYP2D6 activity is largely preserved with aging [31]	PBPK simulation [30]; in vivo probe-drug literature [31]	The modelled decrease and the preserved-activity conclusion come from different methods and are not strictly comparable: the simulation propagates age-related changes in liver mass, microsomal protein and blood flow rather than a change in enzyme activity *per se* [30]	Because the modelled fall is an organ-level rather than an enzyme-level effect, the dominant older-adult modifier for *CYP2D6* is phenoconversion by co-medication, not aging
Flow-limited hepatic metabolism (general)	Metabolism of flow-limited drugs is reduced by approximately 40%, consistent with the reduction in hepatic blood flow in older people [33]	Narrative synthesis of in vivo pharmacokinetic studies [33]	The categorisation of individual drugs as flow- or capacity-limited is not always clean [33]	Explains why high-extraction substrates behave differently from low-extraction substrates such as CYP2D6 probes
Phase II conjugation (UGT)	In vitro metabolism of many capacity-limited drugs is relatively preserved, consistent with preservation of phase I and phase II enzyme content and activity with age [33]. However, this apparent stability at the enzyme level should be distinguished from reductions in hepatic blood flow, liver mass, and net in vivo clearance discussed elsewhere in this section, since overall drug clearance can fall with age even when intrinsic enzyme activity per unit of tissue is largely preserved	Narrative synthesis [31,33]	Drug-by-drug variation is substantial and not quantified as a single effect size [33]. This is a limitation because, without a unified quantitative metric, the direction and magnitude of the age-related effect cannot be reliably generalized across the entire class of UGT substrates, nor can it be safely extrapolated to a specific drug lacking direct pharmacokinetic data. It is important to distinguish this lack of a general age-related adjustment factor from the existence of specific genetic guidelines (e.g., *UGT1A1* and irinotecan), which address genotype rather than chronological age	Glucuronidated substrates are less affected by hepatic aging than high-extraction CYP3A substrates
Intestinal ABC transporters (P-gp, BCRP, MRPs)	Targeted duodenal proteomics identified significant age-related changes in protein abundance, with age coefficients ranging from −0.36 to 0.51: decreased abundance of CES2 (carboxylesterase 2, a Phase I drug-hydrolyzing enzyme), PEPT1 (the main intestinal peptide/peptidomimetic-drug transporter), and villin-1 (a structural marker of mature enterocyte brush-border integrity, included here as an indicator of overall enterocyte differentiation rather than a transporter itself), and increased abundance of the efflux transporters BCRP and MRP1, MRP3 and MRP4 [25]	Targeted mass-spectrometry proteomics of duodenal tissue with multiple regression on age and sex; the study is framed specifically around advanced age rather than the general adult age range [25]	A 2026 review reports the opposite direction, with lower intestinal and hepatic BCRP expression in older compared with young adults [26]. One human study documented roughly 60% lower intestinal ABCB1 expression with aging, constrained by a sample size of 8 and spatial sampling bias [26]. In the microdose cocktail study, changes in dabigatran etexilate exposure—the intestinal P-gp probe—were observed in CKD, not with aging alone [32]	It cannot currently support a directional clinical recommendation
OATP1B1/hepatic OATP (encoded by *SLCO1B1*)	No conclusive age effect: pitavastatin, the OATP1B probe, showed only a trend rather than a definite change [32]. Whole-body PET with 11C-glyburide (*n* = 16) found baseline liver exposure to be 42.6 ± 18.4% higher in females over 50 than in males over 50, consistent with higher blood-to-liver transfer rates [34]	In vivo microdose probe [32]; whole-body dynamic PET-MR [34]	The PET study compared males under 30, males over 50 and females over 50 in groups of 4–7; the demonstrated significant difference was between sexes, not between age groups [34]	Sex is currently the better-evidenced determinant of hepatic OATP function than age. Pitavastatin is therefore a clear example of a substrate whose disposition appears minimally age-dependent. For statins specifically, this means that *SLCO1B1* genotype, and not chronological age, remains the actionable variable for myopathy risk, while the age-related rise in exposure seen for the CYP3A-dependent statins (simvastatin and atorvastatin) is better explained by reduced hepatic blood flow and CYP3A-mediated first-pass metabolism than by the transporter itself [32,33]. The two mechanisms act at different steps and should be assessed separately rather than merged into a single age-plus-genotype statement.

Notes: Values from [30] are outputs of in vitro–in vivo extrapolation coupled with physiologically based pharmacokinetic simulation in five-year age bands, not measured clearances; they are labelled “modelled” wherever they appear. Values from [32] are measured geometric mean ratios with 95% confidence intervals from a prospective microdose study.

**Figure 2:**
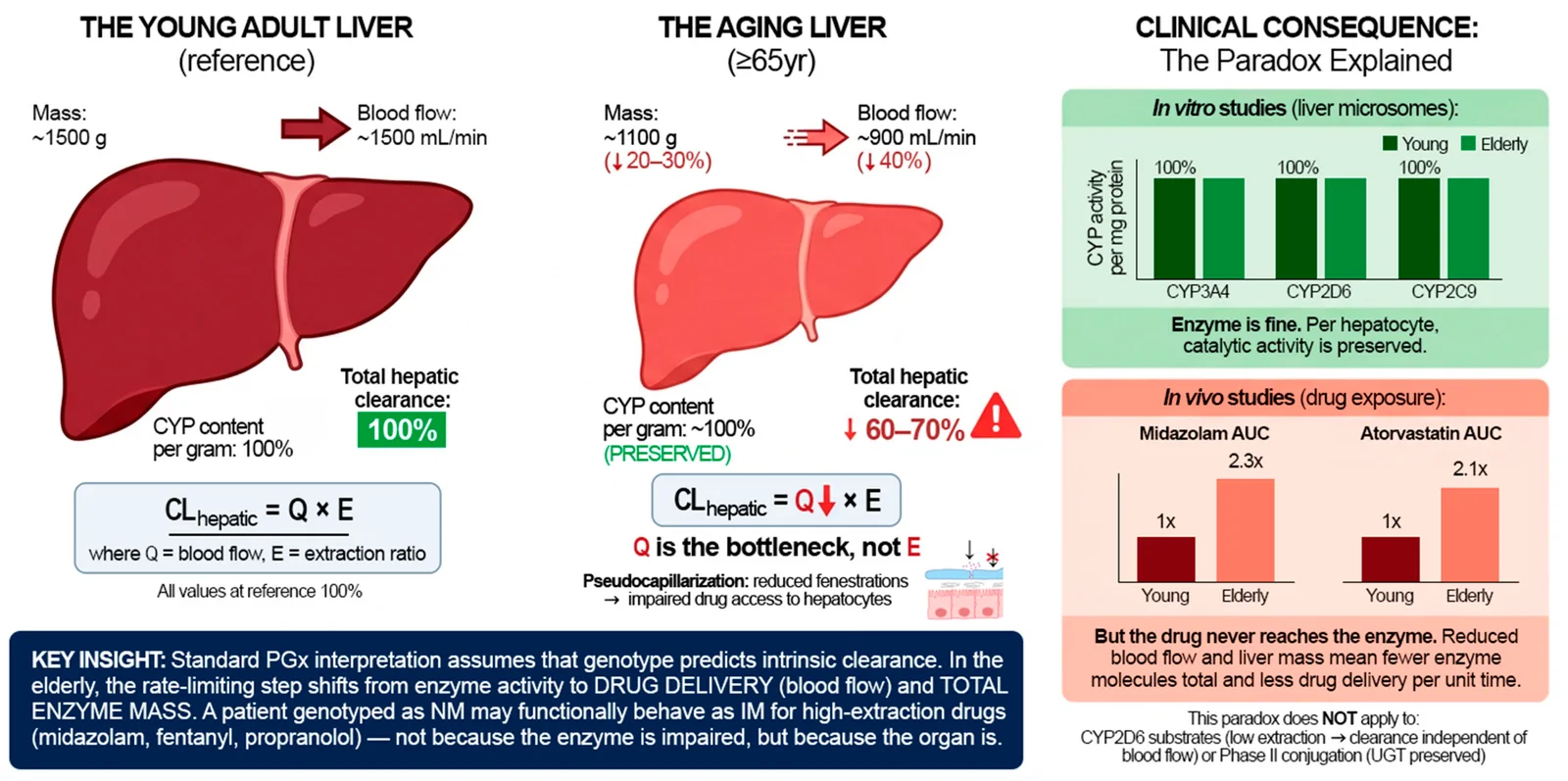
The aging-liver paradox: preserved enzyme activity per gram, reduced clearance in vivo. Comparison of hepatic clearance determinants between young adult and aging livers (≥65 years). While cytochrome P450 activity per gram of liver tissue remains largely preserved with age, total hepatic clearance declines due to reduced liver mass (↓20–30%), decreased hepatic blood flow (↓35–50%), and pseudocapillarization (reduced sinusoidal fenestrations), which impair drug access to hepatocytes. In vitro studies show comparable CYP activity per mg protein between young and old, whereas in vivo studies demonstrate a consistent increase in drug exposure (AUC ratio) for the high-extraction CYP3A4 substrates shown: 2.30 (95% CI 1.70–3.09) for midazolam and 2.14 (1.52–3.02) for atorvastatin, measured in the same microdose probe study [32]. This paradox implies that genotype-predicted normal metabolizer status may functionally manifest as an intermediate metabolizer in the elderly for high-extraction drugs, where the rate-limiting step shifts from enzyme activity to drug delivery (blood flow) and total enzyme mass. This phenomenon primarily does not apply to low-extraction CYP2D6 substrates or Phase II conjugation (UGT).

Molecular aspects of geriatric pharmacotherapy were reviewed by Rzeczycki et al. [6], who emphasized that systemic cytochrome P450 activity, especially in phase I reactions, is significantly reduced with age. As illustrated in Figure 2, this reduction in overall in vivo metabolic capacity is driven primarily by decreases in liver mass and hepatic blood flow, rather than by a loss of intrinsic catalytic activity per hepatocyte (which, as shown in Table 1, is relatively preserved). The analysis covers changes in pharmacokinetics, including the role and regulation of CYP enzymes, whose systemic clearance decreases substantially in older patients despite preserved intrinsic activity. Phase II pathways like glucuronidation appear less affected by age, as seen with drugs such as lorazepam [3]. First-pass metabolism diminishes with age, likely due to decreased liver size and blood flow as well as reduced overall in vivo metabolic capacity of CYP and other biotransformation enzymes [3]. Consequently, the bioavailability of drugs that undergo significant first-pass metabolism, such as opioids and metoclopramide, can substantially increase, necessitating lower initial doses [3,35].

#### 2.1.4. Drug Excretion

Elimination refers to the final process by which a drug exits the body. For most drugs, elimination occurs primarily through the kidneys. Kidney function typically starts to decline around mid-life and continues to decline with age [3,36]. This decline is linked to changes such as reductions in kidney size and filtering capacity. Renal blood flow declines by approximately 10% per decade after age 40 [3]. Glomerular filtration rate declines by 6.3–8 mL/min/1.73 m^2^ per decade after age 30–40 [3,13,37]. Tubular function also declines with age, affecting the secretion and reabsorption of numerous drugs, including organic anions and cations [3,13].

As people age, reduced lean muscle mass can lower serum creatinine despite declining glomerular filtration, making serum creatinine alone an imperfect marker of renal function in older adults [3,38]. Renal function should therefore be assessed using an appropriate estimated glomerular filtration rate (eGFR) and, when relevant to the drug and clinical setting, creatinine clearance; cystatin C-based estimates can provide complementary information when creatinine is potentially misleading. The choice of renal estimate matters because drug-label dosing recommendations may have been derived using different measures. To prevent toxicity from drug accumulation in geriatric patients with decreased renal function, dosage reduction is advised for drugs with a narrow therapeutic index such as digoxin and theophylline [3]. Adjustments in daily doses or dosing frequency are necessary for drugs that heavily rely on renal elimination [3,39].

#### 2.1.5. Integration of ADME Changes: Does One Stage Offset Another?

The preceding sections describe absorption, distribution, metabolism, and excretion as separate processes—an approach that is analytically convenient but can obscure the fact that the net effect on drug exposure depends on how the stages interact. To our knowledge, no study has followed a single drug through all ADME stages in older adults to determine whether changes in one stage offset another; the closest approximation is the microdose cocktail study by Rattanacheeworn et al. [32], which measured multiple pathways in the same participants.

For digoxin, the reduced volume of distribution (from loss of lean body mass) and the reduced renal clearance (from the age-related decline in GFR) act in opposite directions on the elimination half-life, and the two effects are partially offsetting, which is why digoxin dosing in older adults is driven primarily by renal function rather than by age *per* *se* [3,19]. For lipophilic drugs such as diazepam, the increased volume of distribution and the reduced hepatic clearance act in the same direction, producing a substantially prolonged half-life [3]. For high-extraction CYP3A substrates such as midazolam, the age-related fall in hepatic blood flow reduces first-pass metabolism (raising bioavailability) and reduces systemic clearance simultaneously, producing a disproportionately large increase in AUC relative to the change in any single determinant [32,33]. As noted above, a microdose cocktail study [32] found that the age-related increases in exposure were consistent for the main CYP3A probes, although not universally across all transporters (e.g., there was no conclusive change for OATP1B1 or intestinal P-gp), suggesting that compensatory mechanisms between ADME stages are limited in the healthy elderly; in the presence of CKD, the same study found much larger increases in exposure, indicating that organ dysfunction amplifies rather than compensates for age-related changes. The practical implication for pharmacogenomic interpretation is that a genotype-based dose recommendation that was derived from a single-pathway pharmacokinetic study (e.g., CYP2D6 phenotype and metoprolol clearance) may substantially underestimate the total exposure change in an older patient in whom distribution, hepatic blood flow, and renal clearance have all changed concurrently. This is a further argument for combining genotype with measured renal and hepatic function rather than relying on genotype alone.

### 2.2. Pharmacodynamic Changes

Pharmacodynamics describes the effects of drugs on the body [40]. The pharmacological impact of a drug is influenced by the quantity and affinity of target receptors at the site of action, as well as signal transduction and homeostasis regulation [41]. Age-related pharmacodynamic changes are best understood not as a uniform decline in receptor function but as a resetting of the dose–response relationship, in which the same plasma concentration produces a different effect in an older than in a younger patient. A central characteristic of aging is the progressive decline of homeostatic mechanisms, meaning that recovery of physiological function after drug-induced perturbation takes longer, and the frequency of adverse effects increases [19,42].

**Cardiovascular signalling.** Aging is associated with reduced β-adrenergic receptor density and sensitivity, attenuating the chronotropic and inotropic responses to β-agonists such as dobutamine and salbutamol [3,43,44]. Mechanistically, this reflects several converging changes: reduced β-receptor density on cardiomyocytes; uncoupling of the receptor from its Gs-protein, with decreased agonist-stimulated adenylyl cyclase activity and reduced cyclic AMP generation; and impaired downstream protein kinase A signalling [43]. The reduced sensitivity to β-adrenoceptor stimulation appears to be partly a consequence of elevated plasma noradrenaline levels, due to decreased presynaptic α_2_-adrenoceptor activity [3]. Baroreceptor reflex sensitivity declines with age due to reduced arterial compliance and impaired afferent signalling, predisposing older adults to orthostatic hypotension with drugs that reduce peripheral vascular resistance [45]. Drug-related orthostatic reactions occur in 5–33% of older patients, increasing the risk of syncope and falls [45]. This has direct implications for pharmacogenomic interpretation: because the *ADRB1* and *ADRB2* variants most strongly associated with β-blocker response alter receptor signalling at the same node that aging affects, the two effects are not independent, and an age-stratified analysis is required to separate them [PharmGKB].

**Central nervous system.** Geriatric patients exhibit increased sensitivity to central nervous system depressants, including benzodiazepines, opioids, and antipsychotics [45,46,47]. Several structural and functional changes underlie this vulnerability: loss of neuronal substance and reduced synaptic density; impaired brain glucose metabolism; altered neurotransmitter concentrations and receptor density; increased blood–brain barrier permeability; and reduced P-gp-mediated efflux at the blood–brain barrier, which increases brain exposure to substrates independent of plasma concentration [3,48]. Because many of these substrates are also CYP2D6 or CYP2C19 substrates (Section 5), the same drug can be simultaneously affected by a pharmacokinetic genetic variant and by an age-related pharmacodynamic change at the target organ—a distinction that is often conflated when the term “sensitivity” is used loosely.

**Endocrine and metabolic pharmacodynamics.** A characteristic feature of pharmacodynamics in the very old is increased resistance to hormonal agents [42]. Age-related decline in glucose tolerance results from decreased insulin secretion and decreased insulin sensitivity (insulin resistance). Because counter-regulation of glucose is impaired in older adults, the risk of hypoglycaemia caused by sulfonylurea derivatives is increased [42]. This is directly relevant to pharmacogenomic interpretation, since CYP2C9 poor metabolizer status further prolongs sulfonylurea exposure (Section 5) and the pharmacodynamic predisposition to hypoglycaemia and the pharmacokinetic effect of genotype therefore act in the same direction.

**Homeostatic reserve and clinical implications.** Finally, alterations in homeostatic mechanisms—including weakened reflex tachycardia, and disrupted regulation of temperature and electrolyte balance—increase the likelihood of ADRs [41,45]. These changes increase the likelihood of ADRs at doses that are well tolerated in younger patients, and they represent a pharmacodynamic rather than a pharmacokinetic mechanism. The practical implication is that genotype-based dose recommendations derived from pharmacokinetic endpoints alone may underestimate the total effect in an older patient, and that pharmacodynamic monitoring (heart rate, blood pressure, cognitive status, glucose) remains essential even when genotype is known.

## 3. Polypharmacy

**Figure 3:**
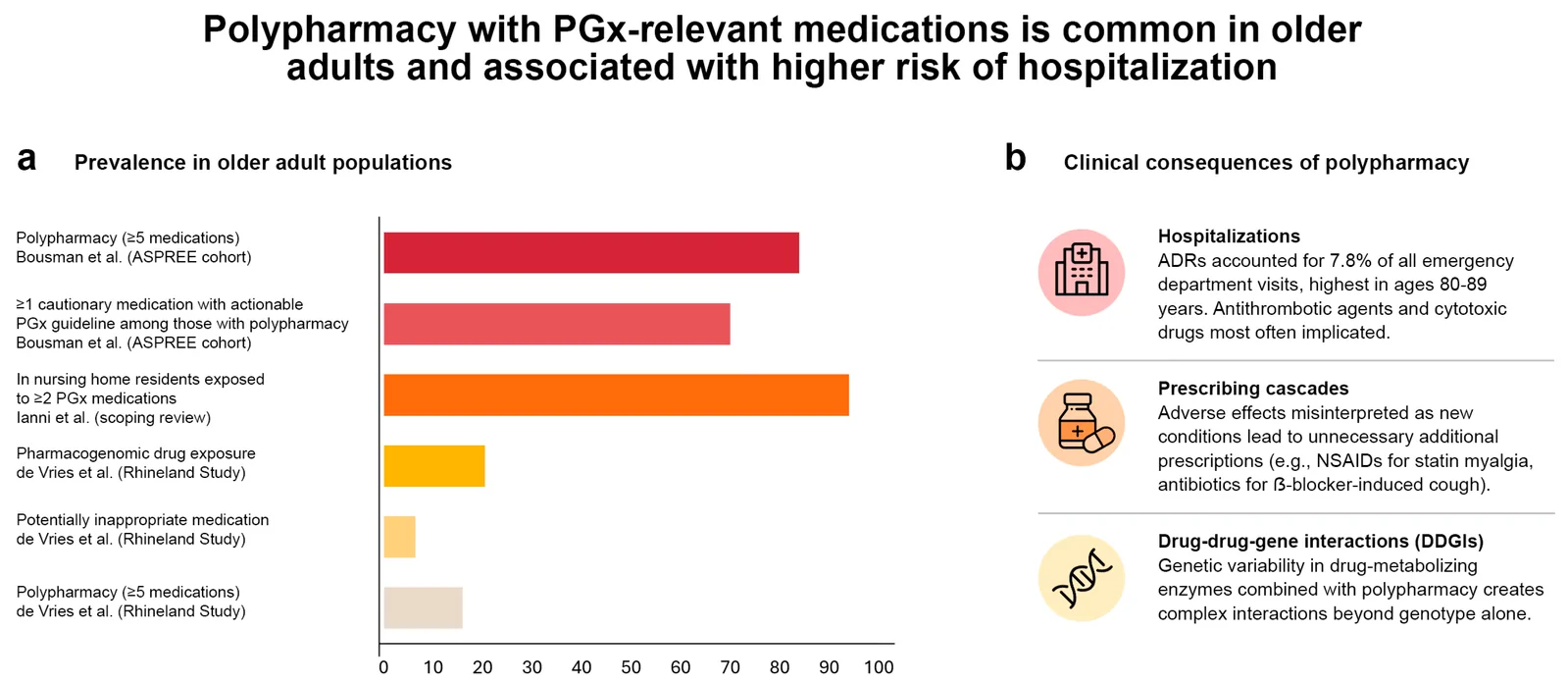
Polypharmacy and pharmacogenomic medication exposure in older adults. (**a**) Prevalence of polypharmacy, potentially inappropriate medication use, pharmacogenomic drug exposure, and exposure to multiple pharmacogenomic-relevant medications. The *x*-axis represents the prevalence (%) of each exposure among the respective source population. Data are based on the ASPREE cohort [7] and a scoping review [5]. The Rhineland Study [1] is included to provide a broad-age community baseline; the bars show its whole-cohort prevalences, while the main text describes the within-study age gradient. Potentially inappropriate medication use in the Rhineland Study was defined using the PRISCUS list (a list-based consensus criterion for older adults), which overlaps with but is not identical to pharmacogenetic actionability. Because the three source populations differ in age range, follow-up window, and medication-count threshold, the bars should be read as separate study-level estimates rather than as a pooled or directly comparable series. (**b**) Major clinical consequences and pharmacogenomic implications of polypharmacy in older adults, including adverse drug reactions (ADRs), prescribing cascades, and drug–drug–gene interactions (DDGIs). Data and concepts are summarised from the cited literature.

Polypharmacy is the norm rather than the exception in geriatric practice [1,5,9,49,50,51] (Figure 3). de Vries et al. [1] examined polypharmacy, potentially inappropriate medication, and pharmacogenomic drug exposure in the Rhineland Study (over 5000 adults), reporting rates of polypharmacy (defined as the regular concurrent use of five or more drugs or prescribed supplements (15.9%)), potentially inappropriate medication (6.4%), and pharmacogenomic drug exposure (20.5%); among participants aged 65 years or older, 54.1% carried at least one of these risk factors, and 27.4% carried two or more, which can be compared with only 16% of those under 65. Longitudinal data from English primary care have confirmed substantial and early-life exposure to pharmacogenomic drugs [52].

The Rhineland Study is particularly informative despite its broad adult age range precisely because it reports the same three exposures inside and outside the geriatric range within a single population and therefore supplies the younger-comparator contrast that geriatric-only cohorts cannot provide: the prevalence of carrying at least one of the three risk factors rises from 16% below 65 years to 54.1% at 65 years and above, and the prevalence of carrying two or more rises to 27.4% [1]. It is this within-study age gradient, rather than the absolute whole-cohort prevalences, that is the relevant observation for the present review.

Potentially inappropriate medication is a list-based rather than a clinical category: a drug counts as potentially inappropriate when an explicit consensus list judges its benefit–risk balance to be unfavourable in older adults, irrespective of the individual patient. It is reported alongside the other two exposures here because the categories overlap without being interchangeable—several of the classes flagged as potentially inappropriate are also pharmacogenetically actionable, in which case the two constraints apply concurrently rather than one substituting for the other. In the Rhineland Study, potentially inappropriate medication was defined by the PRISCUS list, a catalogue constructed specifically around the German drug market and intended for people aged 65 and over; the Beers criteria were not applied because they include agents that are not marketed in Europe [1,53]. The reported rate of 6.4% is therefore a whole-cohort figure spanning an age range extending well below the age at which the list is intended to apply, and it is not directly comparable with estimates generated from the Beers or STOPP/START criteria [1]. Polypharmacy in that cohort was defined as the regular concurrent use of five or more drugs or prescribed supplements [1].

Bousman et al. [7] studied a large cohort of community-dwelling older adults (aged ≥70 years, and ≥65 years for U.S. minority groups) and found that 83.9% experienced polypharmacy (defined as ≥5 medications simultaneously) during a 5-year period. Among those with polypharmacy, 70.0% used at least one cautionary medication with actionable PGx guidelines, whereas cautionary medication use among those without polypharmacy was 66.6%. This narrow gap suggests that, in this cohort, exposure to at least one actionable PGx-relevant medication is driven mainly by the high background prevalence of individual cautionary drugs (such as statins or Proton Pump Inhibitors (PPIs)) prescribed independently of the total medication count. It does not, however, imply that polypharmacy is irrelevant to overall pharmacogenomic risk: the analysis captures exposure to a single cautionary medication, not the cumulative risk of ADRs or the clinical utility of testing, both of which are likely to increase with the number of concurrent medications. Furthermore, among 31 real-world utilisation studies included in a scoping review by Ianni et al., 13 reported exposure to two or more concurrent PGx medications. The three studies restricted to adults aged 65 and over reported rates of 25–94%, with the highest proportion (94%) observed in nursing home residents aged 80.1 ± 10.2 years (*n* = 90) [5]. This underscores the high prevalence of exposure to multiple (≥2) concurrent PGx medications and supports the case for multi-gene panel testing in this population. Ianni et al. also identified that medications most frequently prescribed included pantoprazole (range 0–49.6%), simvastatin (range 0–54.9%), and ondansetron (range 0.1–62.6%) [5].

The clinical consequences of polypharmacy are substantial.

**Hospitalizations:** ADRs are a significant cause of emergency care for older adults. In a retrospective study of a university hospital emergency department (patient age range 16–94 years, mean 63.4), ADRs accounted for 7.8% of all emergency visits [9]. Crucially, the incidence rose steeply with age: while ADRs were observed only infrequently in patients aged 20–49 years, they exceeded 8% of emergency visits in the 50–89-year age range and reached 14% in men and 19% in women aged 80–89 years [9]. This age-dependent gradient demonstrates that vulnerability to ADRs increases with age.

**Link to PGx:** The link between these ADR data and pharmacogenomics is indirect but specific. The drugs most frequently implicated in ADR-related emergency visits—antithrombotics and cytotoxics—are precisely the classes for which actionable pharmacogenetic guidelines exist (*CYP2C9*/*VKORC1* for warfarin, *CYP2C19* for clopidogrel, *DPYD* for fluoropyrimidines, *UGT1A1* for irinotecan) [5,12,54,55]. Notably, the original study [9] provides a quantitative estimate of this link: of the 125 ADR cases, 29 involved drugs covered by CPIC guidelines, but only 2 cases (1.6% of all ADRs) were judged potentially preventable by genotype-guided prescribing (codeine-induced ileus and capecitabine-induced hypokalemia). Fourteen warfarin-related bleeding events were not considered preventable because warfarin had been in use for more than six months in every case, a time when genotype-guided dosing is no longer considered useful. While cytotoxic and antithrombotic agents carry intrinsic toxicity risks at any age, the reduced physiological reserve and higher burden of comorbidity in older adults make the clinical consequences of these events disproportionately severe. Two considerations should temper any inference from these data. First, many ADRs in these classes are dose-independent (e.g., immune-mediated reactions) and would not be prevented by genotype-based dosing. Second, the age-dependent increase in ADR rate observed in the ED data cannot be fully explained by the age-dependent changes in pharmacokinetic exposure documented for most CYP pathways (Section 2.1.3), which suggests that pharmacodynamic and reserve-related factors, not pharmacokinetic ones alone, drive the geriatric ADR burden. This is consistent with the graded conclusion of this review: pharmacogenomic testing is expected to prevent a subset of ADRs in older adults, but its overall contribution to reducing ADR-related emergency care in this population remains modest and should not be overestimated.

**Prescribing cascades:** ADRs are misinterpreted as symptoms of a new condition, leading to unnecessary additional prescriptions [3]. Common examples include prescribing Non-Steroidal Anti-Inflammatory Drugs (NSAIDs) for statin-induced myalgia or antibiotics for cough induced by ACE inhibitors. Prescribing cascades are particularly consequential in older adults because reduced homeostatic reserve and a high baseline medication count make a new drug-induced symptom more likely to be misattributed to an emerging age-related illness rather than recognized as iatrogenic [3].

**Drug–drug–gene interactions (DDGIs):** Genetic variability in drug-metabolizing enzymes, combined with polypharmacy, creates complex interactions that cannot be predicted by genotype alone [51,56].

Pharmacist-led interventions that combine medication review with pharmacogenomic testing illustrate the scale of clinically actionable overlap: in a cohort of 200 pharmacogenomic consultations within the Program of All-Inclusive Care for the Elderly (PACE), in which participants had a mean age of 75.4 ± 9.7 years (range 55.0–99.0; median 75.0, IQR 68.0–83.0), 165 participants carried at least one actionable drug–gene pair, and over one-third of all identified drug–gene pairs were judged clinically actionable, translating into substantial estimated cost avoidance once pharmacists’ recommendations were implemented [57]. Complementary service-implementation work has combined clinical decision-support systems with buccal-swab pharmacogenomic testing for primary-care patients on seven or more medications, aiming to flag multidrug and drug–gene interactions before they cause harm [49]. At the level of individual toxidromes, pharmacists’ recognition of chronic serotonin toxicity in polypharmacy patients has been proposed to benefit specifically from pharmacogenomic data on serotonergic drug metabolism (especially *CYP2D6* and *CYP2C19*), since a patient’s underlying metabolizer phenotype can determine whether an otherwise unremarkable combination of serotonergic agents accumulates to toxic effects [47].

In a small pilot case–control study, frequently hospitalized older adults with polypharmacy (defined as the use of ≥5 prescription medications) had a higher burden of major pharmacogenetic polymorphisms and gene–drug interactions than matched controls [58]. These findings support the hypothesis that pharmacogenetic testing could help identify older adults with polypharmacy at highest risk of hospitalization, although larger studies are needed to confirm causality.

## 4. Role of Pharmacogenetic Variants

Pharmacogenomic testing offers a promising strategy to prevent ADRs and optimize therapy in older adults [5,8,9,10,11,12]. In a longitudinal NHS primary-care analysis of 648,141 patients aged 50–99 years, Kimpton et al. [52] found high exposure to PGx-relevant drugs, with three genes—*CYP2D6*, *CYP2C19*, and *SLCO1B1*—accounting for >95% of pharmacogenomically relevant drug prescriptions, supporting a focused pre-emptive panel design [52].

Lozupone et al. [50] reviewed pharmacogenetics of neurological and psychiatric diseases at older age, concluding that pharmacogenomics is underutilized in geriatric neuropsychiatry despite strong evidence. Pirmohamed [8] provided a comprehensive overview of pharmacogenomics status and future perspectives, emphasizing the gap between genotype-based predictions and observed phenotypes (phenoconversion).

Chen et al. [59] conducted a pharmacogenetic analysis of the model-based pharmacokinetics of five anti-HIV drugs in 74 patients with a median age of 48 years (a general adult cohort, not geriatric-specific), demonstrating that aging significantly influences drug exposure and that pharmacogenetic effects may be modified by age. This underscores the importance of age-adjusted pharmacogenomic interpretation.

Importantly, having an actionable genotype does not necessarily translate into a prescribing change, as the clinical actionability of testing depends on the specific drugs a patient is taking. It is therefore worth separating three quantities that are often reported as though they were one, because they differ by a factor of approximately 2.5 to 3.5 at each step. The first is exposure—the proportion of older adults taking at least one medication with a pharmacogenetic recommendation—which is 69.5% over five years in ASPREE [7], above 43% at a given time in a large Chinese cohort of older adults [60] and 25–94% for exposure to two or more such medications across the studies collated by Ianni et al. [5]. The second is actionability—the proportion in whom genotype and current medication coincide such that a deviation from standard prescribing would be recommended—which falls to 27.5% in the same ASPREE cohort, even though 98.8% of its participants carried at least one actionable genotype and 61.1% carried an actionable *VKORC1* genotype [7]. The third is realised prescribing change—the proportion in whom the pharmacogenetic result actually altered a prescription—and this is the quantity for which geriatric data are weakest: none of the cohorts reviewed here report it directly, and the closest available proxy, antidepressant switching in a Swiss claims dataset of 41,275 patients, indicates that older patients were less likely than younger patients to switch to a drug with an available pharmacogenetic dosing guideline [46]. Carrying an actionable genotype, being prescribed a drug to which it applies, and having prescribing changed as a result are therefore three nested and progressively smaller populations, and any statement about the yield of testing should specify which of them is meant.

Ianni et al. (2025) identified 215 unique medications with pharmacogenetic recommendations prescribed to older adults, of which 82 (38%) were associated with actionable CPIC guidelines (levels A, A/B, or B) [5]. This highlights that the breadth of actionable pharmacotherapy in this population substantially exceeds the scope of single-gene testing approaches.

## 5. Most Significant Pharmacogenes

Population-level data provide context for the scale of PGx actionability in geriatrics. In a large cohort of 13,670 older adults, Bousman et al. found that *VKORC1* (61.1%) and *CYP2C19* (59.6%) were the most frequently observed actionable genotypes, followed by *CYP2B6* (42.6%), *CYP2D6* (41.3%), *CYP2C9* (36.7%), and *SLCO1B1* (29.1%) [7]. These findings align with a larger scoping review of 31 studies, which identified *CYP2D6* (25.6%), *CYP2C19* (18.3%), and *CYP2C9* (11%) as the most frequent genes implicated in actionable drug–gene interactions in older adults [5]. Table 2 summarises the core pharmacogenes relevant to geriatric prescribing, their predicted phenotype frequencies, age-related functional changes, and representative drugs.

Before reviewing individual pharmacogenes, it is worth stating explicitly what this review claims and does not claim about geriatric specificity. It does not claim that any pharmacogene behaves differently in older adults at the level of allele frequency or intrinsic enzyme kinetics—allele frequencies are age-independent, and there is no evidence that a CYP2D6 poor metabolizer genotype produces a different intrinsic metabolic capacity in a 75-year-old than in a 35-year-old. It does claim that the clinical consequences of the same genotype differ systematically with age, for four reasons documented in the preceding sections: (i) the prevalence of exposure to actionable drugs is much higher in older adults [5,7,60]; (ii) the probability of phenoconversion through co-medication is much higher, because polypharmacy is the norm [7,51,56]; (iii) the dose–exposure relationship for a given genotype is shifted by age-related changes in hepatic blood flow, liver mass, body composition, and renal function [32,33]; and (iv) the margin between a tolerated and a harmful exposure is narrower, because of reduced physiological reserve and altered pharmacodynamics [45,53]. These four factors are precisely why the drug classes governed by these genes—antidepressants, antipsychotics and opioid prodrugs for *CYP2D6*; PPIs, clopidogrel and SSRIs for *CYP2C19*; warfarin, NSAIDs and sulfonylureas for *CYP2C9*; and statins for *SLCO1B1*—dominate geriatric prescribing and recur in the Beers and STOPP/START criteria [53]. It is the product of these factors—high exposure, phenoconversion, shifted pharmacokinetics, and narrower safety margins—rather than the size of any individual pharmacokinetic effect, that makes these four genes the rational core of a compact pre-emptive panel for older adults, and that distinguishes their role here from their role in general adult pharmacogenomics. These four pharmacokinetic genes (*CYP2D6*, *CYP2C19*, *CYP2C9*, *SLCO1B1*) form the core of the panel; *VKORC1* is added as a pharmacodynamic target, and HLA-B*58:01 as an immunogenetic marker, yielding a minimum panel of six genes. *ABCB1* is not included because the evidence for its clinical actionability remains insufficient.

The pharmacogenetic phenotype terminology in this review follows the standardized nomenclature agreed by CPIC and the Clinical Pharmacogenetics Working Group and the allele and allele-function definitions maintained by PharmVar (www.pharmvar.org). For the metabolising enzymes CYP2D6, CYP2C19 and CYP2C9, the categories used are ultrarapid (UM), rapid (RM), normal (NM), intermediate (IM) and poor metabolizer (PM), with rapid metabolizer applied only to CYP2C19, for which that category is defined. For *SLCO1B1* the phenotype categories are function-based—increased, normal, decreased and poor function—and are not interchangeable with metabolizer status, because the gene encodes an uptake transporter rather than an enzyme. Throughout, genotype-predicted phenotype denotes the phenotype inferred from diplotype and activity score in the absence of interacting co-medication, whereas measured or observed phenotype denotes a phenotype determined experimentally or clinically, from a probe drug, an endogenous or dietary biomarker, or therapeutic drug monitoring. The distinction is load-bearing rather than cosmetic in this context, because phenoconversion is by definition a discordance between the two and can be demonstrated only where both are available, as in the solanidine-based phenotyping of 88 geriatric patients discussed in Section 5.1 [51].

**Table 2:** Core pharmacogenes relevant to prescribing in adults aged 65 and over.

Gene	Pharmacological Role	Genotype-Predicted Phenotype Distribution	Effect of Aging on Activity	Representative Guideline-Covered Drugs Used in Older Adults
*CYP2D6*	Oxidative metabolism of many antidepressants, antipsychotics, beta-blockers and opioid prodrugs; low hepatic extraction ratio	53.2% predicted normal metabolizers (*n* = 5408) [61] Actionable-genotype frequency 41.3% [7]	Intrinsic activity largely preserved [31]; the modelled 31% fall in desipramine clearance between ages 20–25 and 65–70 [30] reflects liver mass and blood flow rather than enzyme activity. Phenoconversion by co-medication is the dominant modifier [56]	Metoprolol (DPWG), tramadol, codeine, ondansetron, paroxetine, nortriptyline, amitriptyline, tamoxifen (CPIC level A) [5]
*CYP2C19*	Metabolism of proton-pump inhibitors, bioactivation of clopidogrel, metabolism of citalopram/escitalopram and sertraline	39.7% predicted normal metabolizers (*n* = 5408) [61] Actionable genotype in 59.6%—second most frequent gene in ASPREE (*n* = 13,670) [7]	Modelled S-mephenytoin clearance falls 39% between ages 20–25 and 65–70 [30]; capacity-limited pathway, so blood-flow decline contributes less than for high-extraction drugs [31,33]	Clopidogrel, pantoprazole, omeprazole, lansoprazole, citalopram, escitalopram, sertraline, voriconazole (CPIC level A) [5,62]
*CYP2C9*	Metabolism of S-warfarin, most NSAIDs, phenytoin and sulfonylureas	64.8% predicted normal metabolizers (*n* = 5408) [61] Actionable-genotype frequency 36.7% [7]	Modelled S-warfarin clearance falls 36% between ages 20–25 and 65–70 [30]. Direct in vivo probe data in older adults remain limited [31]	Warfarin, celecoxib, ibuprofen, meloxicam, piroxicam, flurbiprofen, phenytoin, fluvastatin (CPIC level A) [5,63]
*VKORC1*	Pharmacodynamic target of vitamin K antagonists; not a metabolic enzyme	Actionable genotype in 61.1%—most frequent gene in ASPREE (*n* = 13,670) [7]	No metabolic aging effect applies. Age enters warfarin dosing as an independent clinical covariate, not through this gene	Warfarin, acenocoumarol, phenprocoumon (CPIC level A) [5]
*SLCO1B1*	Encodes OATP1B1, which mediates hepatic uptake of all statins	Actionable-genotype frequency 29.1% [7] Not reported separately in [61]; refer to the CPIC allele frequency tables	No conclusive age effect on OATP1B1 activity: pitavastatin exposure was not conclusively altered by aging in a microdose cocktail study [32]. Whole-body PET with the OATP probe 11C-glyburide (*n* = 16) identified sex, not age, as the demonstrated determinant of hepatic exposure [34]	Simvastatin, atorvastatin, rosuvastatin, pravastatin, pitavastatin, lovastatin, fluvastatin (CPIC level A) [63]
*CYP3A5*	Together with CYP3A4 determines tacrolimus disposition	Not reported separately in [7] or [61]	CYP3A activity, as a whole, declined markedly with age independently of *CYP3A5* genotype [32]	Tacrolimus (CPIC level A) [5]

Furthermore, 68.2% of participants in the ASPREE cohort reported taking at least one inhibitor or inducer of *CYP2B6*, *CYP2C9*, *CYP2C19*, *CYP2D6*, or *CYP3A5* during the study period. Commonly used inhibitors included esomeprazole (28.7%, CYP2C19 moderate inhibitor), amlodipine (22.9%, CYP3A5 moderate inhibitor), and omeprazole (8.6%, CYP2C19 weak inhibitor). The most common inducers were corticosteroids such as prednisone (10.5%) and betamethasone (7.1%), all of which are weak CYP3A5 inducers [7]. These findings emphasize that phenoconversion—where drug interactions alter metabolic capacity independently of genotype—is likely common in geriatric populations and should be considered alongside genetic testing.

While CYP enzymes represent the most extensively studied pharmacogenes, they are not the only determinants of drug response in older adults. Pharmacogenetic associations have also been described for biologic therapies used in autoimmune diseases, where variants in genes encoding drug targets or immune receptors may influence treatment outcomes. Variants in *FCGR3A* (rs396991) have been associated with response to rituximab, and polymorphisms in *TNF* (rs1800629) may influence response to etanercept in rheumatoid arthritis and other inflammatory conditions [PharmGKB]. While these associations are not yet part of routine clinical guidelines, they highlight the potential for PGx-guided selection of biologic therapies in older adults with autoimmune diseases. It should be noted that the *FCGR3A*–rituximab and *TNF*–etanercept associations described above are general pharmacogenomic findings, established mainly in adult populations not selected for age; age-specific data are currently lacking, and this should be regarded as an extrapolation from general pharmacogenomics rather than geriatrics-specific evidence. These associations are discussed here because rituximab and etanercept are used in indications that are frequently encountered in the age group covered by this review, so these associations will arise in geriatric prescribing; their evidential status, however, remains that of a general adult pharmacogenomic finding, and, on that basis, they are not included in the minimum panel proposed in Section 12.

### 5.1. CYP2D6

*CYP2D6* is arguably the single most consequential pharmacogene in geriatric pharmacotherapy, both because of the breadth of drugs it metabolizes (approximately 25% of all marketed drugs, including antidepressants, antipsychotics, β-blockers, and opioids) [51,64,65,66] and because its functional activity is disproportionately unstable in polypharmacy. In a cohort of 464 genotyped patients undergoing therapeutic drug monitoring (396 aged ≤65 years and 68 aged >65 years), serum concentrations of risperidone and its active metabolite 9-hydroxyrisperidone varied with both age and *CYP2D6* genotype, indicating that the relationship between *CYP2D6* genotype and exposure may vary with age, rather than simply being shifted by a constant factor [65]. Similarly, in 173 patients with polypharmacy (defined as the use of at least seven daily medications) and mean ages ranging from 79.7 to 84.1 years across *CYP2D6* genotype subgroups, prescribed daily doses of CYP2D6-metabolized drugs with hemodynamic effects differed by *CYP2D6* genotype-predicted metabolizer status. However, the study did not demonstrate corresponding differences in measured hemodynamic responses. Rather than negating the role of CYP2D6, this dissociation between drug exposure and clinical effect may be consistent with age-related pharmacodynamic tolerance or compensatory homeostatic mechanisms, which could attenuate the translation of pharmacokinetic differences into measurable clinical outcomes. This highlights the necessity of combining genotype-based dose adjustments with therapeutic drug monitoring and clinical assessment in this population [66]. A population pharmacokinetic model built on 325 older adults with depression (mean age 68.5 ± 6.9 years) treated with venlafaxine (up to 300 mg/day for 12 weeks) found that CYP2D6 metabolizer status affected drug clearance and exposure to both venlafaxine and its active metabolite at both lower and higher dose ranges [64].

Beyond germline genotype, CYP2D6 activity in this population is further destabilized by phenoconversion—a mismatch between genotype-predicted and actually observed metabolic phenotype, driven by comorbidity and, especially, concomitant CYP2D6-interacting drugs. Using a validated dietary biomarker (solanidine) to phenotype CYP2D6 activity directly, Sarömba et al. [51] studied 88 geriatric patients (median age 83, IQR 77–87; median 15 concurrent medications) and found a progressively greater downward shift from genotype-predicted CYP2D6 activity toward the measured functional phenotype as the number of CYP2D6-interacting substrates and inhibitors increased, demonstrating that genotype–phenotype discordance is common in polypharmacy settings and that functional phenotyping provides additional value beyond genotyping alone [51]. Phenoconversion is not restricted to CYP2D6 substrates in the strict sense: a case report of a 68-year-old bilateral lung transplant recipient documented severe, dose-related bradykinesia after tacrolimus (a CYP3A4/5 substrate) was co-administered with two CYP3A4/5 inhibitors (fluconazole and diltiazem), illustrating how drug–drug interactions (DDIs) can convert a genotypically normal metabolizer into a functional poor metabolizer regardless of the gene involved [67].

Phenoconversion is not in itself an age-specific phenomenon—it arises from drug interactions, inflammation and organ dysfunction at any age—and the reason it is treated as central here is quantitative rather than mechanistic. Three conditions required for genotype–phenotype discordance co-occur far more often in older adults than in younger ones: exposure to at least one interacting inhibitor or inducer, which reached 68.2% of participants in ASPREE [7]; a number of concurrently interacting agents sufficient for the shift to become clinically meaningful, with the downward shift in measured CYP2D6 activity scaling with the number of interacting drugs in a cohort whose median medication count was 15 [51]; and reduced physiological reserve, which makes the resulting rise in exposure more likely to cross a toxicity threshold. The consequence is that in older adults, the genotype-predicted phenotype is a systematically biased estimate of the metabolic capacity actually available, biased towards overestimating it, whereas in a younger population taking fewer concurrent medications, the probability of clinically significant phenoconversion may be lower, although this depends on the specific drugs, doses, and clinical states. This is a difference in the prior probability and the expected magnitude of discordance rather than in the underlying biology, and it is on that basis—not on a claim that the mechanism is unique to aging—that medication reconciliation is placed ahead of genotype interpretation in Table 3.

**Table 3:** Practical recommendations for pharmacogenetic interpretation in older adults.

#	Recommendation	Supporting Evidence	Strength of Support	Reference
1	Reconcile current medication for cytochrome P450 inhibitors and inducers before translating genotype into a predicted phenotype	In the ASPREE cohort, 68.2% of participants reported use of at least one inhibitor or inducer of a cytochrome P450 enzyme linked to a prescribing guideline during the trial. In an acute aged-person mental health cohort (*n* = 137), accounting for phenoconversion increased the predicted frequency of CYP2D6 intermediate metabolizers by 11.7% at admission and 16.1% at discharge and that of CYP2C19 intermediate metabolizers by 13.1% and 11.7%. Of 120 prescriptions of 19 medications with actionable recommendations at admission, 50 (42%) carried an actionable recommendation before phenoconversion, and 60 (50%) carried one after.	Direct—two cohorts with quantified effect	[7,56]
2	Prioritise a compact pre-emptive panel centred on *CYP2D6*, *CYP2C19*, *CYP2C9*, *SLCO1B1*, *VKORC1*, and HLA-B*58:01	Across 31 real-world utilisation studies, the genes most frequently implicated in actionable drug–gene interactions were *CYP2D6* (25.6%), *CYP2C19* (18.3%) and *CYP2C9* (11%). In cohorts restricted to adults aged 65 and over, *CYP2C19* was the predominant gene among the ten most-prescribed actionable drugs. In ASPREE, *VKORC1* (61.1%) and *CYP2C19* (59.6%) were the most frequent genes carrying actionable genotypes, and statins, NSAIDs and proton-pump inhibitors were the most-used cautionary medication classes.	Direct	[5,7]
3	Consider pre-emptive multigene panel testing when the expected clinical benefit, medication exposure, and feasibility of implementation justify testing, while acknowledging that outcome-based evidence in older adults remains limited	A total of 98.8% of ASPREE participants carried at least one actionable genotype, with a mean of 3.04 per participant. Among studies restricted to adults aged 65 and over, 25–94% of patients were exposed to two or more medications with pharmacogenetic recommendations; the highest proportion was in nursing home residents.	Direct for exposure prevalence; indirect for comparative clinical utility over reactive testing	[5,7]
4	Treat frailty as a priority indication rather than a reason to defer testing	In 59,973 NHS hospital admissions of patients aged 65 and over, models incorporating the number of guideline-covered medications were the best fit for length of stay and for repeat admission specifically in the high-frailty stratum; in the low- and intermediate-frailty strata, models with and without that variable performed comparably. Frailty carried the largest coefficient of any predictor (9.64 for high frailty in the unplanned-admission model).	Indirect—the study had no genotype data and the authors state this explicitly. It supports prioritising frail patients for testing; it does not demonstrate that guideline doses require further downward adjustment in frailty	[68]
5	Interpret genotype alongside renal function rather than in isolation	In the microdose cocktail study, older adults with CKD had markedly greater exposure than older adults without it for the same probes: atorvastatin AUC ratio 4.15 (2.98–5.79) versus 2.14 (1.52–3.02), and midazolam 2.90 (2.16–3.88) versus 2.30 (1.70–3.09). Altered exposure of the intestinal P-gp probe dabigatran etexilate was observed in the CKD group specifically.	Direct, though from a single small study	[32]
6	Do not transfer adult CPIC dose recommendations to patients over 75 without accounting for reduced CYP3A capacity	Midazolam AUC was 2.30-fold higher and atorvastatin AUC 2.14-fold higher in healthy older adults than in healthy young adults, in participants unselected for actionable genotype. Metabolism of flow-limited drugs is reduced by approximately 40% in older people, consistent with the reduction in hepatic blood flow.	Direct for the magnitude of the CYP3A effect; the extrapolation to a specific dose adjustment is expert opinion	[32,33]
7	Re-examine the pharmacogenetic interpretation at every change of therapy rather than treating the report as a one-off result	A total of 83.9% of ASPREE participants reported polypharmacy (five or more concurrent medications). In a Danish nursing home cohort of 141 residents aged 65–98 years (65–74: *n* = 22; 75–84: *n* = 51; 85–98: *n* = 68), the most frequent drug interaction flagged with a warning was the combination of a proton-pump inhibitor with clopidogrel, a recognised phenoconversion mechanism.	Direct for the prevalence data; the recommendation to re-review is a reasonable inference from it	[7,69]
8	State the limits of the report explicitly, including that genotype does not capture inhibitors and inducers, renal function, hepatic function or frailty status	No dataset quantifying the effect of this reporting practice was identified.	Expert opinion—no supporting data available	—
9	Where a strong inhibitor can be withdrawn, do so before phenotyping or before initiating a sensitive substrate	In contrast to genotype, phenoconversion is a reversible pharmacological effect; the quantitative effect of withdrawal on predicted phenotype in older adults remains unquantified.	Expert opinion, mechanistically grounded—no outcome dataset identified	[56] for the magnitude of phenoconversion only
10	Use therapeutic drug monitoring in preference to genotype-only interpretation for narrow-therapeutic-index drugs in patients with several concurrent risk factors	No study has quantified the proportion of pharmacokinetic variability explained by genotype specifically in older adults.	Expert opinion—no supporting data available	—

### 5.2. CYP2C19

*CYP2C19* polymorphisms are clinically important in older adults across at least two major drug classes: PPIs and SSRIs. For omeprazole—the PPI most sensitive to *CYP2C19* genotype among commonly used agents—Na et al. [70] demonstrated that *CYP2C19* polymorphisms affected omeprazole pharmacokinetics in 15 healthy elderly subjects aged 65–73 years, with poor metabolizers having substantially higher drug exposure, extending earlier work showing that age itself alters the relationship between *CYP2C19* genotype and omeprazole pharmacokinetics.

For escitalopram, a combined physiologically based pharmacokinetic (PBPK)-modelling and clinical study on 88 elderly patients with major depressive disorder (aged ≥65 years) found that simulated escitalopram exposure was 2.1-fold higher in CYP2C19 poor metabolizers than in normal metabolizers; consistent with this, dose-normalized trough concentrations in the clinical cohort were 1.6-fold higher in poor metabolizers (*p* = 0.0132) [71]. Based on these findings, the authors proposed that a 10 mg/day dose may be appropriate for poor metabolizers, while normal and intermediate metabolizers who do not respond at 10 mg/day could be titrated up to 20 mg/day—a more granular, phenotype-specific recommendation than the blanket dose ceiling currently advised for elderly patients generally [71]. This finding is notable because it directly challenges a one-size-fits-all geriatric dosing cap by showing that the “safe” dose for one metabolizer subgroup may be subtherapeutic for another.

The influence of *CYP2C19* phenotype on drug response was also examined by Jang et al. [72], who applied PBPK modelling to compare fluoxetine pharmacokinetics between younger adults (18–65 years) and elderly population (65–98 years), quantifying age-related differences in fluoxetine pharmacokinetics and evaluating the influence of *CYP2C19* phenotype.

In a large population-based cohort, *CYP2C19* was the second most frequent actionable gene (59.6%); across all actionable genes, an estimated 27.5% of participants used at least one medication (e.g., PPIs, clopidogrel) for which a deviation from standard prescribing would be recommended [7]. PPIs were used by 16.6% of participants; overall, 7.9% of all participants used PPIs and carried an actionable *CYP2C19* genotype [7]. Across multiple studies, pantoprazole (range 0–49.6%), omeprazole (range 0–34.8%), and citalopram (range 0–17.8%) were among the most frequently prescribed CYP2C19 substrates [5].

### 5.3. CYP2C9 and VKORC1

Note on evidence level: The *CYP2C9*–warfarin and *VKORC1*–warfarin associations reviewed in this section are general adult pharmacogenomic findings, established predominantly in mixed-age cohorts and incorporated into CPIC and DPWG guidance without age stratification [5]. They are not, in themselves, geriatric-specific: allele frequencies do not change with age, and the genotype–dose relationship for warfarin is qualitatively the same in a 45-year-old and an 85-year-old. What is geriatric-specific is the context: warfarin remains one of the most frequently prescribed actionable drugs in older adults [5,7]; the consequences of over-anticoagulation (major bleeding, falls) are disproportionately severe in this age group; and the co-administration of CYP2C9 inhibitors or inducers—which phenoconverts a genotypic normal metabolizer into a functional poor metabolizer—is more common in older adults because of polypharmacy. Crucially, the evidence for a geriatric-specific magnitude of the genotype effect (as distinct from its clinical importance) remains limited: while dedicated cohorts in the very elderly have identified additional genetic contributors (such as *EPHX1*) and genotype-guided trials have demonstrated benefit in elderly Chinese patients, age-stratified effect estimates against younger comparators are largely unavailable.

*CYP2C9* and *VKORC1* are key determinants of warfarin dosing [12,73]. Lin et al. [73] genotyped 16 variants across *VKORC1*, *CYP2C9*, *CYP4F2*, and *EPHX1* in very elderly, frail Han-Chinese patients (mean age 80.6 ± 4.6 years); a multivariable model combining *VKORC1* rs9923231, *CYP2C9* rs1057910, *EPHX1* rs2260863, *CYP4F2* rs2189784, and body surface area explained 26.9% of warfarin dose variability—notably including *EPHX1*, a gene outside the standard *VKORC1*/*CYP2C9* pairing, as an independent contributor in this age group [73].

In a randomized controlled trial of 507 Chinese elderly patients with nonvalvular atrial fibrillation (mean age 71.41 ± 6.86 years in the genotype-guided group and 70.63 ± 6.11 years in the control group), genotype-guided warfarin dosing significantly improved the percentage of time in the therapeutic INR range (TTR) compared with standard clinical dosing (70.80 ± 24.39% vs. 53.44 ± 26.73%; absolute difference 17.36%, 95% CI: 11.82–22.89; *p* < 0.001). The cumulative incidence of ischaemic stroke was also significantly lower in the genotype-guided group (2.39% vs. 6.82%; HR 0.22, 95% CI: 0.065–0.77; *p* < 0.05), while hemorrhagic events did not differ significantly between groups [12].

Unlike *CYP2D6* and *CYP2C19*, where age-related and phenoconversion effects have been characterized in reasonably large cohorts, *CYP2C9* evidence specific to older adults is comparatively sparse and often entangled with *CYP2C19* in combined analyses. A large naturalistic therapeutic drug monitoring study—857 serum concentrations from 252 patients on valproic acid (mean age 45.1 years, range 16–89), genotyped for both *CYP2C9* and *CYP2C19*—examined age, sex, and genotype together as covariates of the dose-adjusted steady-state concentration (C:D ratio), reflecting the reality that in routine geriatric psychiatric and neurological practice these two genes are rarely evaluated in isolation from one another or from age [74].

This entanglement matters for interpretation: because *CYP2C9* and *CYP2C19* jointly influence valproic acid clearance, and age independently affects the same pharmacokinetic parameters, isolating a pure “*CYP2C9*-in-the-elderly” effect from a single study is difficult, and stronger gene-specific claims for *CYP2C9* in geriatric populations would benefit from additional dedicated cohorts, as evidence from dedicated geriatric cohorts remains limited and further studies are needed to establish the independent contribution of *CYP2C9* in older adults.

Among community-dwelling older adults, *VKORC1* (61.1%) and *CYP2C9* (36.7%) are among the most frequently observed actionable genotypes [7]. Although warfarin use is declining with the introduction of DOACs, *VKORC1* and *CYP2C9* genotyping remains relevant for older adults on warfarin therapy and for predicting sensitivity to other vitamin K antagonists. Warfarin was prescribed to 1.1–11.1% of older adults across included studies [5].

### 5.4. SLCO1B1 and Statins

Note on evidence level: The *SLCO1B1*–statin associations are general adult pharmacogenomic findings. They are not age-specific, but their clinical relevance is amplified in older adults because statins are among the most frequently used cautionary medications [7], and the consequences of statin-induced myopathy (immobility, deconditioning) are disproportionately severe. However, age-related changes in statin exposure are driven primarily by reduced hepatic blood flow rather than by a loss of OATP1B1 function [32,33].

Statin pharmacogenomics in older adults centers on *SLCO1B1*, the hepatic uptake transporter gene most strongly linked to statin-associated myopathy risk, though much of the applied literature frames this within the broader problem of statin DDIs in an aging, polypharmacy-exposed population rather than as an isolated genetic effect [75,76,77]. Bellosta and Corsini [77] catalogued the range of statin DDIs and their mechanisms as statin use expanded in the elderly, while Damiani et al. [75] focused specifically on elderly patients (over 75 years old), noting that comorbidity burden and polypharmacy compound the risk that a DDI will precipitate statin-associated myopathy or hepatotoxicity in this age group.

Direct pharmacogenetic evidence pertaining to older adults is more limited, and this is where Srimatimanon et al. [76] filled a specific gap: a pharmacokinetic study on 50 healthy Thai adults, with median ages of 30 years (range 23–40) in the young-adult group and 65 years (range 60–77) in the elderly group, examined the effect of age on plasma exposure to simvastatin and its active metabolite, simvastatin acid, explicitly accounting for key *SLCO1B1* variants known to affect statin disposition, and situated its findings against a meta-analysis of published data from other ethnic populations together with an exploratory pharmacogenetic screen of additional disposition-related genes [76]. The study found that aging significantly increased simvastatin C_max_ while SLCO1B1 decreased function was associated with higher simvastatin acid exposure.

Read together, these three sources illustrate a common pattern across genes discussed in this section: age and genotype are rarely investigated as fully independent factors, but rather as compounding variables that jointly determine statin exposure and myopathy risk in the aging patient.

Statins were the most frequently used cautionary medications in a recent large cohort of older adults, with 29.3% of participants reporting use of atorvastatin, rosuvastatin, or simvastatin [7]. Among statin users, approximately 28–30% carried actionable *SLCO1B1* or *CYP2C9* variants for which dose adjustment or alternative medication would be recommended to reduce the risk of musculoskeletal symptoms [7,63]. Simvastatin was one of the most frequently prescribed PGx medications across 31 studies, with prescribing rates ranging from 0% to 54.9% [5].

### 5.5. ABCB1

*ABCB1* (encoding P-gp) evidence pertaining to older adults spans both drug efflux at the intestinal/renal level and drug-induced neurotoxicity. In 128 patients aged 80 and older (median age 87.5, IQR 83.0–90.0) with nonvalvular atrial fibrillation, *ABCB1* (rs1045642, rs4148738) genotype did not significantly alter overall steady-state rivaroxaban trough concentration or prothrombin time, but carriers of the rs4148738/rs1045642 TT genotype experienced clinically relevant non-major bleeding more frequently than CC carriers—indicating that in the very old, *ABCB1* genotype may matter more for a specific safety endpoint (bleeding) than for average pharmacokinetic exposure [78].

Tanabe et al. [79] found that paclitaxel-induced sensory peripheral neuropathy is associated with an *ABCB1* single nucleotide polymorphism (1236 TT genotype) and older age in Japanese patients (median age 50 years, range 25–75), suggesting that *ABCB1* genotyping may help predict neuropathy risk.

### 5.6. HLA

Beyond the metabolism- and transport-focused genes above, *HLA* variants are the dominant pharmacogenetic risk factor for a distinct and life-threatening category of adverse reaction—severe cutaneous adverse reactions (SCARs)—that disproportionately affects older patients treated for gout. In a Thai case–control study on 86 allopurinol-induced SCARs (19 DRESS, 67 SJS/TEN) compared with 182 allopurinol-tolerant controls—with a median age of 68 years (range 28–84) overall, with 68.6% of SCAR cases aged over 60 years—HLA-B*58:01 carriage was associated with a markedly elevated risk of both DRESS (OR 149.2) and SJS/TEN (OR 175.0), with female sex being an additional independent risk factor (OR 4.6); overall mortality among SCAR cases was 11.4% [80]. Given that allopurinol is a first-line urate-lowering therapy commonly initiated in older adults with gout and reduced renal clearance, this gene stands apart from the others reviewed in this section in that its clinical actionability rests on pre-treatment screening to prevent a rare but often fatal reaction, rather than on titrating a dose within a tolerated range.

Note on evidence level: While the HLA-B*58:01–allopurinol association is a general adult finding with strong evidence across all age groups, its clinical consequences (SCARs) disproportionately affect older patients with gout and reduced renal clearance. Because the clinical actionability rests on pre-treatment screening to prevent a rare but often fatal reaction, the absolute benefit of testing is likely to be highest in this vulnerable population.

## 6. Cardiovascular Pharmacogenomics

Pharmacogenomic testing in cardiovascular disease has been extensively studied in older adults [11,75,77,78,81]. For example, variants in *ACE* (rs1799752, rs4646994) have been associated with response to ACE inhibitors, and polymorphisms in *ADD1* (rs4961) may influence hydrochlorothiazide efficacy [PharmGKB]. Božina et al. [11] reviewed the use of pharmacogenomics in elderly patients treated for cardiovascular diseases, providing evidence for *CYP2C9*, *VKORC1*, *SLCO1B1*, and *CYP2C19* in warfarin, clopidogrel, and statin therapy.

Chai et al. [81] investigated metoprolol population pharmacokinetics in 42 older Chinese patients with CKM syndrome aged ≥60 years (60–93), stratified into younger-old (60–74 years, *n* = 21) and older-old (≥75 years, *n* = 21) groups, demonstrating joint effects of rs1065852 (*CYP2D6*) and clinical scores on clearance, supporting integrated pharmacogenomic and clinical modelling for beta-blocker dosing.

Bellosta and Corsini [77] and Damiani et al. [75] reviewed statin interactions and *SLCO1B1*-related myopathy risk in elderly patients. Božina et al. [11] provided evidence for *CYP2C9*, *VKORC1*, *SLCO1B1*, and *CYP2C19* in warfarin, clopidogrel, and statin therapy.

The therapeutic classes most frequently studied in relation to pharmacogenetic prescribing in older adults were psychotropic (*n* = 29), analgesic (*n* = 29), and cardiovascular (*n* = 27) medications, reflecting the high prevalence of these drug classes in geriatric practice and their substantial pharmacogenetic evidence base [5].

### 6.1. Antihypertensives

Hypertension is a significant risk factor for cardiovascular disease, stroke, and renal disease, and its prevalence notably increases with age, affecting approximately 65% of geriatric patients [82]. The pathophysiology of hypertension in the elderly is complex and multifactorial, characterized by increased total peripheral vascular resistance, decreased arterial compliance, reduced cardiac output, increased blood pressure variability due to age-related baroreceptor function decline, and decreased blood flow with dysregulated auto-regulation in critical organs [83,84].

All antihypertensive drugs can predispose older patients to symptomatic orthostatic hypotension, postprandial hypotension, falls, and syncope. The adverse effects vary depending on the specific antihypertensive drugs used, their dosages, the presence of co-morbidities, and potential DDIs [85,86]. Delirium linked to β-blocker use may manifest through symptoms like confusion, disorientation, agitation, aggression, and visual and auditory hallucinations [87]. Calcium channel blockers also have notable adverse effects; for example, verapamil is primarily linked to bradyarrhythmia and constipation, while nifedipine is associated with hypotension, peripheral oedema, skin rash, and tachycardia [88].

### 6.2. Diuretics

Pharmacogenomic status: There are no actionable gene–drug pairs. Age-related changes in renal tubular function can influence how drugs affect the kidneys, particularly in conjunction with organic acid diuretics like furosemide and bumetanide [89,90]. Diuretic therapy in geriatric patients requires careful management due to their increased susceptibility to fluid and electrolyte imbalances, such as hypokalaemia, hyponatraemia, hypomagnesaemia, and volume depletion [91,92]. Additionally, diuretics can trigger delirium through dehydration and electrolyte disturbances [93]. Diuretics can cause hyponatraemia, and SSRIs (metabolized by CYP2C19 and CYP2D6) can independently cause SIADH. Co-administration may therefore increase the risk of clinically significant hyponatraemia [53], representing a DDI that is further modulated by CYP2C19 and CYP2D6 genotype and is therefore not captured by any single-gene test.

### 6.3. Digoxin

Pharmacogenomic status: There are no actionable guidelines from CPIC/DPWG; the relevant determinants are renal and transporter-mediated. Digoxin is effectively absorbed in the gastrointestinal tract, but in older adults, the rate of absorption is slightly delayed (time to peak concentration increases from ~45 min to ~105 min), and the elimination half-life is prolonged from approximately 38 h to 69 h [94]. Reduced lean body mass lowers the volume of distribution, so loading doses should be reduced by approximately 20% [42]. The dominant mechanism of age-related toxicity risk is renal: digoxin is eliminated largely unchanged by the kidney, and the age-related decline in GFR (Section 2.1.4) directly prolongs its elimination half-life. Additional factors contribute, including temporary dehydration and the use of NSAIDs (including COX-2 inhibitors), which are prevalent in this age group [95]. Electrolyte imbalances such as hypomagnesaemia, hypercalcaemia, hypernatraemia and hypokalaemia can alter the myocardial effects of digoxin even when serum concentrations are within the therapeutic range, and thyroid dysfunction changes both its volume of distribution and clearance [95]. Digoxin is also a P-gp substrate; amiodarone and verapamil inhibit P-gp and raise digoxin concentrations, whereas macrolides such as clarithromycin increase digoxin bioavailability by inhibiting gut bacteria that inactivate digoxin in 10–15% of patients [95]. The *ABCB1* variant rs1045642 has been associated with altered digoxin pharmacokinetics in healthy volunteers [96]. There is no validated genotype-based dosing guideline for digoxin, and the clinically actionable variable in older adults remains renal function together with a review of P-gp-inhibiting co-medication. This is an example of a drug where pharmacogenomic testing is not the appropriate intervention, and where the correct geriatric action is therapeutic drug monitoring and renal dose adjustment.

### 6.4. Statins

Pharmacogenomic status: Actionable gene–drug pairs exist for statins (*SLCO1B1*, *CYP2C9*). In geriatric patients, statins are generally well tolerated, with most adverse effects being mild and short-lived [97]. Research indicates that the safety profiles of atorvastatin and simvastatin are comparable in patients younger and older than 65 years who have stable coronary disease. However, monitoring levels of creatine kinase and liver enzymes is essential when using statins, as these medications can lead to rhabdomyolysis and myopathy in both elderly and younger individuals [98,99]. From a pharmacokinetic standpoint, most statins are substrates of the hepatic uptake transporter OATP1B1 (encoded by *SLCO1B1*). As discussed above (Table 1), age-related reductions in hepatic blood flow, rather than a primary age-related loss of OATP1B1 transporter function itself, appear to be the more plausible driver of the increased statin exposure observed in older adults. *SLCO1B1* genotype contributes a further, largely age-independent source of interindividual variability in statin exposure and myopathy risk [63,77]. Thus, in older adults, the actionable variable for myopathy risk is the *SLCO1B1* genotype (and renal/hepatic function), rather than chronological age alone.

Statins have been consistently shown to reduce cardiovascular events in both younger and older populations. For patients aged over 65 years, statin therapy reduces the risk of major cardiovascular events by 19%, which is comparable to the 22% reduction seen in younger individuals [100]. However, the benefits of statin treatment typically become evident after at least one year of continuous use, and elderly individuals, due to their shorter life expectancy and higher burden of comorbidities, may derive fewer benefits from statin therapy compared to younger populations [101]. The benefit–risk ratio must be carefully evaluated in geriatric patients, who often have more complex health profiles.

### 6.5. Anticoagulants

Pharmacogenomic status: The status is actionable for warfarin (*CYP2C9*, *VKORC1*, *CYP4F2*); there are no established actionable pharmacogenomic guidelines for direct oral anticoagulants (DOACs). Advanced age is linked to platelet dysfunction, reduced synthesis of coagulation factors, and increased fragility of blood vessels [102]. Administering anticoagulant therapy to prevent stroke in the elderly is particularly challenging due to their heterogeneity, which includes higher rates of comorbidities, polypharmacy, frailty, and cognitive decline, along with functional and psychosocial concerns [103].

From a pharmacogenomic standpoint, the shift toward DOACs has narrowed the population in which *CYP2C9* and *VKORC1* genotyping may inform warfarin initiation. The 2023 AGS Beers Criteria recommend avoiding initiation of warfarin for non-valvular atrial fibrillation in older adults when a DOAC is an appropriate alternative [53]. Pharmacogenetic algorithms may inform initial warfarin dosing in selected patients; however, their role in patients already stable on warfarin is more limited, and INR monitoring remains essential.

For DOACs, renal function is a major determinant of exposure and dosing, rather than an established pharmacogenomic marker. Dabigatran and, to a lesser extent, edoxaban undergo substantial renal elimination; reduced renal clearance may increase exposure and necessitate dose adjustment or avoidance, depending on the drug-specific prescribing criteria (Section 2.1.4). The *ABCB1* variant rs1045642 has been associated with bleeding risk during rivaroxaban treatment in patients aged 80 years and older [78], but the evidence is insufficient to support genotype-guided prescribing. Routine pharmacogenetic testing is therefore not currently recommended to guide DOAC selection or dosing in older adults.

## 7. Psychotropic Drugs and CYP2D6/CYP2C19

Psychotropic drugs are among the most commonly prescribed medications in older adults, and their metabolism is heavily dependent on *CYP2D6* and *CYP2C19* [10,46,50,53,64,65,71,72,104]. Psychotropic medications are frequently used to treat mental health conditions such as depression and anxiety as well as to manage behavioural and psychological symptoms of dementia [105,106]. The use of psychotropic medications tends to increase with age, with up to 63% of residents in care homes across Western Europe prescribed at least one psychotropic drug; in a study of 4,478 residents aged ≥60 years (mean age 86.6 ± 7.6 years in women and 82.2 ± 8.8 years in men), 48.2% had known dementia [107]. This high rate of use is also observed among geriatric patients in community settings [108].

van der Schans et al. [10] conducted the CYSCE trial, a pragmatic randomized controlled trial on *CYP2D6* screening in older patients (mean ages 70.2 ± 7.3, 68.5 ± 5.1, and 71.8 ± 7.2 years across study arms) starting therapy with nortriptyline or venlafaxine. Men et al. [64] demonstrated that CYP2D6 phenotype significantly influences venlafaxine pharmacokinetics in older adults with depression. Molden et al. [65] found that aging significantly increases dose-adjusted serum concentrations of risperidone active moiety in patients with known *CYP2D6* genotype. Jang et al. [71,72] used physiologically based pharmacokinetic modelling to optimize assessment of escitalopram and fluoxetine response in geriatrics, reporting that CYP2C19 phenotype significantly influences drug exposure.

Psychotropic pharmacotherapy is where *CYP2D6* and *CYP2C19* pharmacogenetics converge most consequentially with age, because these are precisely the drug classes (SSRIs, SNRIs, tricyclics, antipsychotics) where single-gene effects are most pronounced. For venlafaxine, CYP2D6 metabolizer status affects drug clearance and exposure to both the parent drug and its active metabolite at both lower and higher dose ranges [64]. For risperidone, serum concentrations of the drug and its active metabolite 9-hydroxyrisperidone vary with both age and CYP2D6 genotype, as detailed in Section 5.1 [65]. For escitalopram, as detailed in Section 5.2, PBPK modelling and clinical data demonstrate significantly higher drug exposure in CYP2C19 poor metabolizers, challenging the standard geriatric dose ceilings [71]. The same research group extended its PBPK approach to fluoxetine and found that steady-state exposure was approximately twofold higher in older adults, whereas CYP2D6 phenotype affected parent-drug but not active-moiety exposure, leading the authors to conclude that CYP2D6-guided dose adjustment is unlikely to be needed for fluoxetine in this population [72]. Whether this pharmacogenetic knowledge changes real-world prescribing is only partially answered by the literature. A Swiss claims-data study of 41,275 patients on escitalopram found that only 6.4% switched to a different antidepressant, and among switchers, just 35.4% moved to a drug with an available PGx dosing guideline; critically, elderly patients were less likely than younger patients to switch to a PGx-guided alternative, despite prior work showing that CYP2C19 poor metabolizers switch away from escitalopram 3.3 times more often than normal metabolizers [46]. This suggests that even where the pharmacogenetic signal for treatment failure is well established, older patients are the group least likely to benefit from it in routine switching behaviour—an implementation gap distinct from, and arguably more concerning than, the biological effect itself.

Direct trial evidence on whether *CYP2D6* testing improves outcomes in geriatric psychiatry is limited. The CYSCE trial—the only pragmatic randomized controlled trial of pre-emptive *CYP2D6* screening in older adults starting nortriptyline or venlafaxine—found no significant difference in time to reach adequate drug levels between the genotype-informed and control arms (DG-I vs. DG-C: *p* = 0.663). A statistically significant difference was observed only when the genotype-informed arm was compared with a non-randomized external control group (*p* = 0.004), while the randomized control arm showed only a non-significant trend (*p* = 0.087). No general effect of genotype information was observed on secondary outcomes, including quality of life and severity of depression. The authors concluded that their results do not support *CYP2D6* screening to accelerate dose adjustment in this population [10]. This null result is an important counterweight to the mechanistic and pharmacokinetic evidence: even for a gene with strong biological plausibility and clear dose–exposure relationships, translating that into a measurable clinical benefit via testing has not been consistently demonstrated in older patients.

### 7.1. Benzodiazepines

Benzodiazepines, being lipid-soluble, tend to have prolonged half-lives in geriatric individuals due to their accumulation in fat tissues. This extended duration of action, combined with the increased sensitivity of geriatric patients to sedative-hypnotics, can lead to delirium [53,104]. Advancing age is linked to heightened sensitivity to the central nervous system effects of benzodiazepines. For instance, diazepam can induce sedation at lower doses and plasma concentrations in geriatric patients [104,109]. The adverse effects of benzodiazepines, such as dizziness, ataxia, drowsiness, and impaired psychomotor function, tend to become more pronounced with age [104]. Research has shown that geriatric individuals taking benzodiazepines with a long elimination half-life face an increased risk of falls, a higher likelihood of hip fractures, and a greater chance of being involved in motor vehicle accidents due to sedation [110,111,112]. According to Beers’ criteria, benzodiazepines as a class should generally be avoided in older adults, as shorter-acting agents are not safer than long-acting ones [53].

### 7.2. Antidepressants

Depression stands out as a leading cause of disability globally and significantly contributes to the overall burden of disease [113]. In geriatric populations (predominantly aged ≥60 years), depression has a pooled prevalence of 31.74% (95% CI: 27.90–35.59), making it one of the most common psychiatric disorders in this age group, posing serious risks for both disability and mortality [114]. In geriatric patients, the manifestation of depression is often complicated by cognitive issues, as symptoms such as memory difficulties, distress, and anxiety can overshadow the underlying depression [114,115].

Tricyclic Antidepressants: While effective, tricyclic antidepressants cause a range of side effects due to muscarinic receptor blockade, including dry mouth, sweating, tachycardia, urinary retention, blurred vision, postural hypotension, and confusion [116]. Of particular concern is postural hypotension, which can lead to serious outcomes such as sudden drops in blood pressure, increasing the risk of hip fractures [117,118]. Among tricyclic antidepressants, nortriptyline and desipramine tend to be better tolerated by older adults [116], although both remain classified as potentially inappropriate in the 2023 AGS Beers Criteria because of their anticholinergic activity. Nevertheless, second-generation antidepressants are generally favoured for geriatric patients and those with heart disease, as they have fewer side effects and are less toxic in overdose situations [119].

Selective Serotonin Reuptake Inhibitors: SSRIs and SNRIs are generally considered easy to administer, yet approximately 35% of geriatric patients diagnosed with depression are prescribed antidepressants at low-intensity regimens [120]. Although fluoxetine is generally avoided in older adults because of its markedly prolonged half-life and potential for DDIs, it is still prescribed in this population [121], and 30–40% of patients treated with fluoxetine do not achieve an adequate therapeutic response [122]. High-quality evidence shows that paroxetine has strong anticholinergic properties and carries a significant risk of causing sedation and orthostatic hypotension, making it unsuitable for use in geriatric patients [53]. Instead, several safer alternatives are recommended for this population, including citalopram, escitalopram, sertraline, venlafaxine, mirtazapine, and bupropion. Citalopram was one of the most frequently studied CYP2C19 substrates, with prescribing rates ranging from 0% to 17.8% [5].

Atypical Antidepressants: Mirtazapine has not been extensively studied in the context of treating late-life anxiety disorders, but its favourable side-effect profile and minimal potential for DDIs make it a recommended option for geriatric patients [119]. Mirtazapine can be particularly beneficial for geriatric adults experiencing anxiety alongside sleep disturbances or appetite loss. Its sedative properties can help improve sleep, while its appetite-stimulating effects can address weight-loss issues [119]. Bupropion, an aminoketone, is an effective and well-tolerated antidepressant for geriatric patients, though its main side effects include insomnia, agitation, and headache, with the most severe being a reduced seizure threshold [123].

### 7.3. Antipsychotic Drugs

Antipsychotic drugs are commonly used to manage psychiatric disorders in geriatric patients. Regulatory agencies issued warnings in the mid-2000s regarding the use of atypical antipsychotics in people with dementia due to an increased risk of death and stroke in this population [124]. Similarly, cohort studies have demonstrated an association between the use of typical antipsychotics and a heightened risk of mortality in geriatric individuals [124,125]. The use of antipsychotic medications, particularly clozapine and olanzapine, has been linked to an increased risk of treatment-induced diabetes mellitus and dyslipidaemia [126]. Both conventional and atypical antipsychotics have been associated with an increased risk of sudden death and pneumonia, likely due to their anticholinergic effects leading to complications such as swallowing difficulties. Other serious risks, such as deep venous thrombosis, have also been reported [126].

### 7.4. Mood Stabilisers and Antiepileptics

Mood-stabilising drugs are commonly prescribed to prevent the recurrence of depression and to manage and treat bipolar disorder [127,128]. Lithium, commonly used to treat bipolar disorder and mania, requires careful plasma concentration monitoring, particularly in geriatric patients [129]. Since lithium is metabolised and excreted by the kidneys and kidney function tends to decline with age, dosages should be reduced for elderly patients [130]. Recent longitudinal studies of long-term care residents with dementia reveal a noteworthy trend: while there has been a 6% decline in antipsychotic prescribing, there has been a significant increase in the use of both sedative and non-sedative antidepressants, as well as a small increase in the use of mood stabilisers [131,132].

The incidence and prevalence of epilepsy are highest among the elderly, nearly twice that of children, and it continues to increase with age [133]. Selecting appropriate antiepileptic drugs for elderly patients is crucial, given the pharmacokinetic and pharmacodynamic changes they experience, as well as their increased susceptibility to DDIs due to polypharmacy. Geriatric patients generally require lower doses of antiepileptic drugs compared to younger adults [134]. Newer antiepileptic drugs tend to be more suitable for elderly patients due to their lower risk of side effects and fewer drug interactions. Traditional antiepileptic drugs like carbamazepine, phenytoin, and phenobarbital are enzyme inducers, which can reduce the efficacy of medications such as anticoagulants, antidepressants, and cardiovascular drugs. In contrast, valproate is an enzyme inhibitor and may increase the concentration of other drugs. Newer antiepileptic drugs like lamotrigine and levetiracetam, with minimal drug interactions, may be more appropriate for geriatric patients on multiple medications [133].

## 8. PGx and Risk of Falls

Falls are among the most consequential ADRs in older adults, and their pharmacogenetic dimension runs through several genes already discussed [45,67,135,136]. A dedicated review identified falls as a distinct ADR category in the elderly, noting that pharmacogenetic variability can contribute to drug-induced falls through impaired elimination in patients with inherited deficiency of CYP2C9, CYP2C19, or CYP2D6 [45]. Interestingly, a subsequent prospective population-based study explicitly designed to test this hypothesis (ActiFE-Ulm, *n* = 1377 community-dwelling older adults, median age 74.5 years in fallers and 74.1 years in non-fallers, 1-year follow-up) found that use of CYP2C19-metabolized drugs was associated with falls, but the genotype-predicted metabolizer phenotype itself was not—a dissociation that complicates the straightforward “poor metabolizer → higher exposure → higher fall risk” narrative and suggests that drug selection and exposure route may matter more than germline genotype alone for this particular outcome [137].

NSAIDs appear to be a major contributing factor to falls. Walker et al. reported a 10-fold increased likelihood of falling with NSAID use (including low-dose aspirin) in 62 hospitalized patients aged ≥65 years matched with 62 non-fallers [138]. A meta-analysis linked NSAID use to an increased risk of falls, with an unadjusted odds ratio of 1.21 [139]. Polypharmacy is also a key risk factor for falls in the elderly [140]. Interestingly, a 2017 study by Zia et al. showed that the use of ≥2 fall risk-increasing drugs, rather than polypharmacy (defined as the regular use of ≥5 prescription drugs) alone, was a significant predictor of falls in participants aged ≥65 years (*n* = 202 fallers, *n* = 156 controls) [141].

Argevani et al. [67] described tacrolimus-induced bradykinesia secondary to phenoconversion in an elderly post-bilateral lung transplant patient, highlighting that drug-induced movement disorders can increase fall risk. James et al. [135] documented nimodipine-induced junctional bradycardia in an elderly patient with subarachnoid hemorrhage, demonstrating that cardiovascular ADRs can precipitate falls (CYP3A5*3/*3 genotype, predicting poor CYP3A-mediated metabolism). Ji et al. [136] presented a case of carbamazepine-induced paroxysmal dysarthria and ataxia in an elderly patient, highlighting that carbamazepine can induce specific neurotoxicity even at standard doses in susceptible elderly individuals.

Although NSAIDs have been implicated in falls and fractures in observational studies, the magnitude of this association varies across populations and study designs, and data specific to older adults are heterogeneous. Further research is needed to clarify the extent to which NSAIDs and other analgesics contribute to falls in geriatric populations and whether PGx-guided prescribing can mitigate this risk.

## 9. Oncology Pharmacogenomics in Older Adults and Transplantation

Older adults with cancer face unique pharmacotherapeutic challenges, including higher rates of comorbidities, polypharmacy, frailty, and altered drug disposition [142,143,144,145,146]. Oncologic pharmacotherapy concentrates several of the risks already discussed in this review—regarding narrow-therapeutic-index drugs, DDIs, and drug–gene interactions—in a population that is disproportionately older and comorbid. The evidence here splits into genuinely pharmacogenetic findings, non-genetic risk-prediction tools, and drug-interaction studies that are germane to safe prescribing but do not involve genotyping; distinguishing between these matters, since not all “toxicity risk” evidence in oncology is pharmacogenetic in nature.

**Genetic evidence.** In gastric cancer patients treated with fluoropyrimidine–platinum chemotherapy (median age < 59 vs. ≥59 years), standard *DPYD* genotypes used in European clinical guidelines occur at a frequency below 0.7% in the Latin American population studied, making them largely unusable as a risk-stratification tool in that population; the authors instead built a combined SNP-plus-clinical-parameter nomogram to predict grade ≥ 3 overall toxicity [143]. In 408 patients with lung cancer receiving platinum-based chemotherapy, polymorphisms in several miRNA genes were associated with treatment-related toxicity: rs2042553 in miR-5197 with overall severe toxicity (dominant model OR 1.80, 95% CI 1.16–2.80), rs2043556 in miR-605 with severe hepatotoxicity (OR 2.51, 95% CI 1.12–4.14), and rs895819 in miR-27a with gastrointestinal toxicity specifically in patients older than 56 years [144]. Tanabe et al. [79] found that paclitaxel-induced sensory peripheral neuropathy is associated with *ABCB1* polymorphism and older age. Mueller-Schoell et al. [145] used pharmacometric simulation to demonstrate that obesity alters endoxifen plasma levels in breast cancer patients (pooled dataset median age 55 years, range 22–95), with implications for older patients.

Tamoxifen, a widely used adjuvant therapy for hormone receptor-positive breast cancer, is metabolized to its active metabolite endoxifen primarily by CYP2D6. Polymorphisms in *CYP2D6* resulting in poor metabolizer status are associated with reduced endoxifen concentrations and potentially decreased treatment efficacy [PharmGKB]. *CYP2D6* genotyping is recommended by CPIC for tamoxifen therapy, though its clinical utility in older adults with breast cancer remains an area of active investigation.

**Non-genetic geriatric-oncology risk tools.** Two widely used clinical risk scores for chemotherapy toxicity—CARG and CRASH—were tested for external validity in a prospective cohort of 248 older patients aged ≥70 years referred for pretherapeutic geriatric assessment, and both performed poorly as predictors of severe toxicity in that external cohort [142]. This null result is a useful counterpoint to genetic approaches: neither purely clinical/functional scores nor (as shown in earlier sections) genotype alone has yet proven to be a reliable, portable predictor of toxicity risk in older cancer patients, suggesting that combined models (as attempted in [143]) may be a more promising direction than either approach in isolation. Complementing risk-scoring approaches, digital/wearable remote-monitoring tools such as GeRI aim to capture functional decline and toxicity signals continuously in the home setting rather than at scheduled clinic visits, though this specific study addressed usability and participatory design rather than toxicity prediction accuracy [146]. Davis et al. [147] provided guideline clinical insights on opioid conversion in adults with cancer. Importantly, that consensus guideline is based on a systematic review and Delphi process; it does not have a direct pharmacogenetic focus, though opioid conversion is indirectly relevant to CYP2D6 metabolism of codeine and tramadol.

**DDGIs in cancer supportive care.** Morgans et al. [148] discussed managing drug interactions with enzalutamide in patients with prostate cancer. Enzalutamide, used for prostate cancer that disproportionately affects men over 65, is a moderate inducer of CYP2C9 and CYP2C19 and a strong inducer of CYP3A4; because these men commonly receive concurrent cardiovascular medication, the authors specifically note that co-administration with warfarin reduces warfarin exposure via CYP2C9 induction and should either be avoided or accompanied by closer INR monitoring [148].

Sadeghi et al. examined co-prescription of low-dose methotrexate and trimethoprim-sulfamethoxazole and the 30-day risk of death among older adults. In a population-based cohort of 3204 older adults aged ≥66 years on low-dose methotrexate, co-prescription of trimethoprim-sulfamethoxazole (versus a cephalosporin) did not increase 30-day mortality (0.87% vs. 0.94%; RR = 0.93) but did increase the risk of hospitalization (RR = 1.49) and infection (RR = 2.78). Although this study does not directly evaluate a pharmacogenetic mechanism, it illustrates the importance of DDIs in older adults, which may interact with PGx factors to influence toxicity risk [149]. This is a reminder that not every clinically important interaction risk in this population is pharmacogenetic, and that non-genetic DDIs remain an important, complementary source of preventable harm alongside the gene-based mechanisms emphasized elsewhere in this review.

Cheung and Tang [150] reviewed personalized immunosuppression after kidney transplantation, discussing the role of pharmacogenomics (CYP3A5, tacrolimus) in optimizing dosing for elderly transplant recipients.

## 10. Real-World Utilization of PGx Drugs in Older Adults

Real-world utilization of medications with pharmacogenetic recommendations in older adults has been examined in several studies [1,5,52,60]. The review highlights significant opportunities for pharmacogenomic-guided prescribing optimization.

The scale is substantial and remarkably consistent across health systems. In a large Chinese population study of older adults (average age 74 years), over 43% were prescribed medications with CPIC Level A actionable pharmacogenetic biomarkers—predominantly *CYP2C19*- and *SLCO1B1*-associated drugs such as clopidogrel and statins—and an estimated 36.5% of those exposed could potentially benefit from genotype-guided dose adjustment [60]. This converges closely with the NHS primary-care finding discussed in the “Role of Pharmacogenetic Variants” Section, where *CYP2D6*, *CYP2C19*, and *SLCO1B1* together governed over 95% of pharmacogenomically relevant prescribing [52], with the scoping review identifying 215 pharmacogenetically relevant medications in real-world use among older adults, 82 of them carrying actionable recommendations [5]—together indicating that pharmacogenetically actionable exposure in this age group is the rule rather than the exception across at least three distinct health systems and populations.

This high background exposure creates the practical context for testing uptake, which itself lags behind exposure. Lee et al. [151] explored facilitators and barriers to the adoption of pharmacogenetic testing in an inner-city population of 120 patients (mean age 55 ± 14 years, 39.2% male, 69.2% African American). Notably, that study was conducted in an antithrombosis clinic setting, where patients were at elevated bleeding risk; despite this risk profile, awareness, knowledge, and willingness to pay for pharmacogenetic testing varied considerably, highlighting the need for education and community engagement to overcome barriers such as perceived negative impact of results and test utility concerns [151].

**Figure 4:**
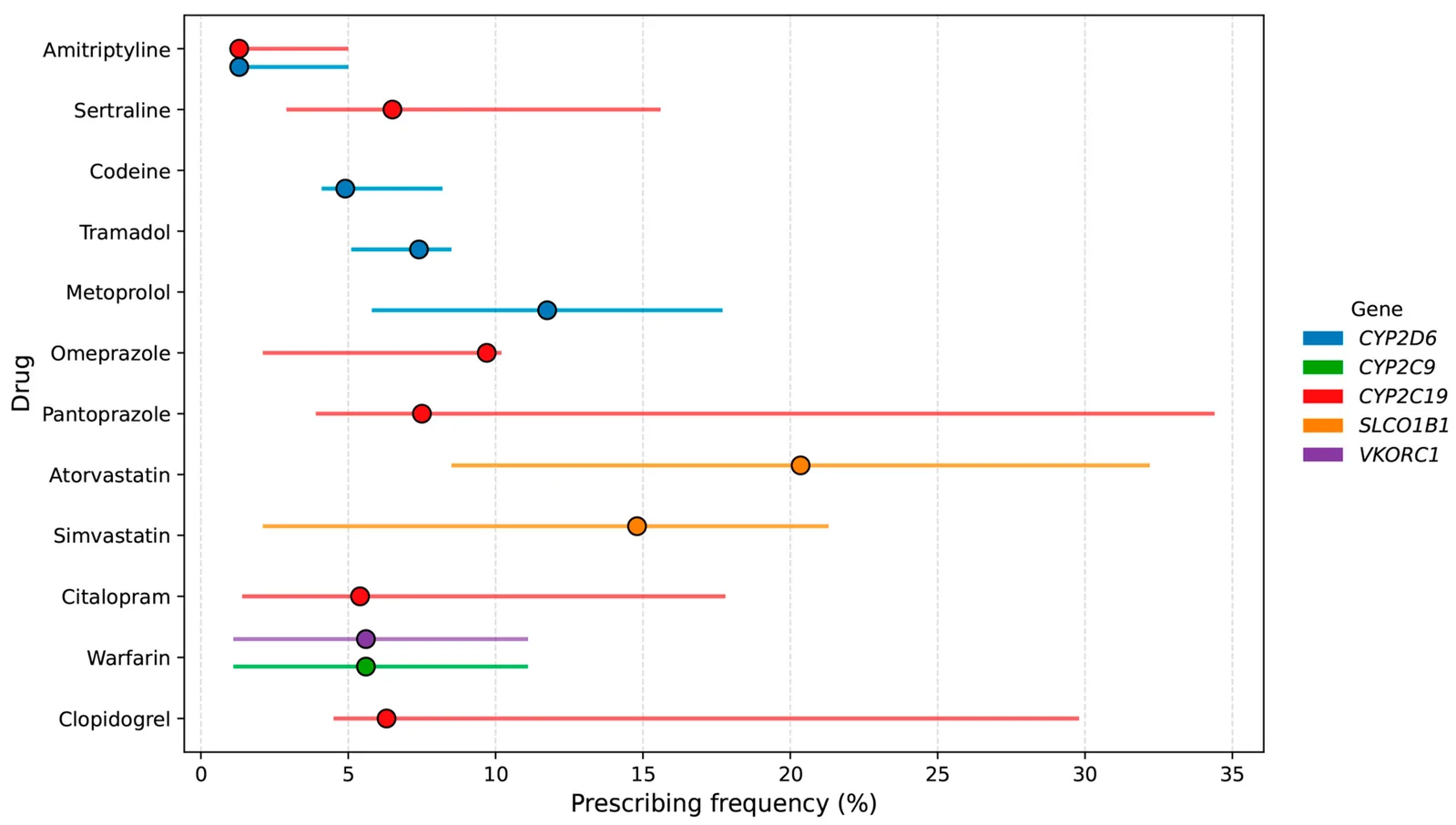
Prescribing frequency of pharmacogenetically actionable drugs in patients ≥ 65 years across five cohort studies. For each drug, the horizontal line represents the range of prescribing rates (min–max) reported in studies with separate data for adults ≥ 65 years [5]. The dot indicates the median rate. Colors correspond to the associated gene; drugs with multiple genes (Warfarin, Amitriptyline) are shown separately for each gene with a slight vertical offset. Notes: Data for Atorvastatin and Metoprolol lacked a published median; their dot positions represent the mean of the range (20.35% and 11.75%, respectively).

Among exclusively older adult cohorts (≥65 years), the most frequently prescribed actionable medications included simvastatin (range 2.1–21.3%), clopidogrel (range 4.5–29.8%), and pantoprazole (range 3.9–34.4%). The genes primarily associated with these medications were *CYP2C19* (*n* = 7) and *CYP2D6* (*n* = 3) [5] (Figure 4). Read together with the sections below on pre-emptive versus reactive testing and barriers to implementation, this pattern—high actionable drug exposure paired with uneven testing uptake—frames pre-emptive testing not as a speculative future capability but as a response to an already-existing, well-quantified prescribing reality.

## 11. Pre-Emptive vs. Reactive Testing

Pre-emptive pharmacogenetic testing is often proposed as advantageous over reactive testing in older adults [8,151] on the grounds that it allows the genotype to be known before the first exposure rather than after an ADR. The rationale is cumulative rather than singular: Pirmohamed [8] frames genotyping cost as no longer the binding constraint on implementation, shifting the practical question from “can we test” to “when should we test”. Given that over 40% of older adults already carry actionable gene–drug exposure at any given time [60] and that this exposure compounds with polypharmacy rather than occurring in isolation, a reactive strategy—testing only after an ADR or treatment failure has already occurred—forfeits the opportunity to prevent the first such event, which in an older, more physiologically vulnerable patient may carry disproportionately severe consequences (as in the HLA-B*58:01/allopurinol SCAR data, where mortality reached 11.4% [80]).

At the same time, the strongest available randomized evidence on reactive-versus-pre-emptive dosing for a specific drug pair is more equivocal than this rationale alone would suggest. As detailed in Section 7, the CYSCE trial found no significant difference in time to reach adequate drug levels between the genotype-informed and randomized control arms (*p* = 0.663); a statistically significant difference was observed only against a non-randomized external control, and the authors concluded that their results do not support CYP2D6 screening to accelerate dose adjustment [10]. This tempers the strength of any blanket recommendation: the case for pre-emptive testing is strongest in cases where the potential harm from a first exposure is severe or irreversible (*HLA-B* SCARs, tacrolimus/CYP3A phenoconversion causing falls) and least clearly established in cases where the outcome of interest is simply reaching an adequate therapeutic drug level somewhat faster, as in CYSCE [10]. Testing strategies, in other words, may need to be tailored to the severity of the downstream event being prevented rather than applied uniformly across all gene–drug pairs—a nuance the facilitators-and-barriers literature suggests is not yet reflected in how testing is actually adopted in practice [151].

Lee et al. [151] found that facilitators of pharmacogenetic testing included providing further information about the test, elaborating on its benefits for predicting treatment efficacy, and patients’ trust in their providers, while barriers included concerns about negative consequences of results and perceived lack of utility among patients whose medications were already working. Kimpton et al. [52] demonstrated that exposure to pharmacogenomic drugs commences early in adulthood, suggesting that pre-emptive testing could provide lifetime benefits. Zhao et al. [60] estimated that a substantial proportion of older adults could potentially benefit from genotype-guided dose adjustment, although direct evidence of reduced ADRs from testing remains limited. Taken together, these strands support a graded rather than a blanket recommendation. Expressed on the four levels used throughout this review, the biological plausibility of pre-emptive testing in older adults is high. Pharmacokinetic evidence is strong for CYP3A (based on measured microdose data) and moderate for CYP2C19 (based on physiologically based modelling, with limited direct in vivo data), whereas for CYP2D6 the evidence for age-related changes is weak (intrinsic activity is largely preserved) even though genotype-predicted effects are strong. Pharmacogenetic actionability is established by existing CPIC and DPWG guidance [5,62,63], which is, however, not age-stratified, and demonstrated clinical utility in older adults specifically remains confined to a small number of gene–drug pairs in which a first exposure can cause severe or irreversible harm. For example, the strong association between HLA-B*58:01 and allopurinol-induced SCARs supports pre-treatment screening, but prospective outcome trials in older adults are still needed to confirm the clinical utility of routine genotyping. We therefore state the recommendation conditionally: pre-emptive panel testing is justified now when the avoidable event is severe and irreversible, and it should be described as a reasonable but not yet outcome-proven strategy in cases where the endpoint is dose optimisation alone. Cost-effectiveness in a geriatric population specifically—in which remaining life expectancy, competing mortality and the horizon over which a single result can be reused all differ from the working-age populations in which most economic models were constructed—has not been formally established and is identified here as a research gap rather than offered as an argument for testing.

Having established both the theoretical rationale and the current limitations of randomized evidence for pre-emptive testing, we now return to concrete recommendations for interpreting pharmacogenetic results in older adults. Table 3 summarises evidence-based guidance on key aspects such as phenoconversion, panel selection, frailty, renal function, and the limits of genotype-only interpretation, with explicit grading of supporting evidence.

In addition to genotype-guided dosing, clinicians must consider how pharmacogenetically actionable drugs intersect with established criteria for potentially inappropriate prescribing in older adults. Table 4 maps this overlap against the AGS Beers Criteria^®^ for the most commonly prescribed drug classes, highlighting both reinforcing and independent constraints.

## 12. Barriers to Implementation and Future Perspectives: Multi-Gene Panels

### 12.1. Barriers to Implementation

Several barriers limit the widespread adoption of pharmacogenetic testing in geriatric practice [5,13,151]. Cost, clinician education, and lack of guidelines are significant barriers to implementation [5]. Lee et al. [151] identified, in an inner-city population, patient concerns about negative consequences of results and a perceived lack of utility among those whose medications were already working. Schlender et al. [13] found that integrating pharmacogenomics into clinical workflows requires overcoming structural barriers, notably electronic health record integration and clinical decision support. Bain et al. [152] proposed a framework for medication risk mitigation in which health care systems, universities, and researchers collaborate to enable practice-based research and implementation of pharmacogenomic services.

The barriers documented across this review fall into three types rather than a single generic “lack of awareness” problem. Patient-level barriers: even in a population with elevated bleeding risk, who stood to gain the most from testing, awareness and willingness to pay varied [151]. System-level barriers: PGx-actionable prescribing is already pervasive (215 relevant medications, 82 actionable [5]), yet it has not been matched by routine testing infrastructure, which implies that the bottleneck lies in clinical workflow and reimbursement rather than in identifying which drugs matter. Knowledge-translation barriers: Streamlining pharmacotherapy for older adults requires integrating pharmacogenetic, pharmacokinetic, and comorbidity information simultaneously [13], which a fragmented “one gene, one drug” testing model does not achieve.

Three further barriers take a specific form in geriatric care, and stating them concretely shows what would have to change.

**Turnaround time.** It is the binding constraint wherever the prescribing decision cannot wait. A result returned within days is compatible with pre-emptive testing at a scheduled contact (a comprehensive geriatric assessment, a memory clinic visit, admission to a nursing home), but not with starting an antiplatelet agent after an acute coronary event or an antidepressant on the day of hospital discharge, which is where a substantial part of geriatric prescribing is actually initiated. This is an argument for testing at a non-urgent point of contact and storing the result for reuse, not for faster assays.

**Cost-effectiveness.** Analyses for this population are scarce. Although the cost of genotyping itself is decreasing [8], results from younger adults cannot simply be transferred. A single panel is amortised over fewer remaining years of prescribing in an 85-year-old than in a 55-year-old, but the per-year density of actionable prescribing is higher and the averted events (a fall with hip fracture, a major bleed, an admission for delirium) are more costly. The direction of the net effect therefore cannot be assumed and requires explicit modelling in geriatric cohorts.

**Clinical decision support.** A rule set that fires on genotype alone is insufficient: a useful alert must combine the genotype with the current medication list, renal function, and the explicit prescribing criteria summarised in Table 4 and must refire when any of these criteria change, not only when the result is filed. We emphasize the need for further evaluation of such CDS approaches in geriatric settings.

### 12.2. Multi-Gene Panels and Future Perspectives

The breadth of actionable PGx medications in older adults exceeds the scope of single-gene testing [5]. Multi-gene panels therefore offer a comprehensive approach to personalized therapy in this population [6,8,59]. Pirmohamed’s framing of pharmacogenomics as a maturing field with decreasing genotyping costs [8], together with molecular perspectives on how the drug-handling machinery itself changes with age [6], supports panel-based, age-integrated testing as a more realistic implementation target than sequential single-gene testing. Rzeczycki et al. [6] further noted that multi-omics approaches (transcriptomics, proteomics, metabolomics) may improve understanding of age-related variability in drug response.

A cohort of 74 adult HIV-infected patients on combination antiretroviral therapy (atazanavir/ritonavir or efavirenz with tenofovir/emtricitabine) illustrates the point. A model integrating pharmacogenetic variants with chronological age and pharmacokinetic data, analysed by ANOVA, multiple linear regression, and Random Forest, was needed to characterize how aging and multiple gene variants jointly shaped exposure to the five antiretroviral drugs [59]. Older patients are exposed to several drugs governed by several genes, each with a comparatively modest effect, and comorbidity modifies drug clearance independently of genotype. Testing built around single gene–drug pairs will therefore systematically underexplain the variability seen in practice.

At a minimum, a panel should cover *CYP2D6*, *CYP2C19*, *CYP2C9*, *SLCO1B1*, *VKORC1*, and HLA-B*58:01—the genes with the strongest evidence base reviewed in Section 4 and Section 5 [5,62,63]. Such a panel is likely to be more clinically relevant than single-gene testing in a population at the highest risk of polypharmacy, where patients rarely present with a single actionable variant in isolation [5]. Its cost-effectiveness in geriatric cohorts, however, remains to be demonstrated (Section 12.1).

Combining multi-gene panels with clinical decision support of the kind described above, as well as performing testing pre-emptively at a non-urgent point of contact, represents a promising implementation model [13,152]. Prospective evaluation of whether this combined approach actually reduces ADRs in geriatric services remains a key priority for future research.

## 13. Materials and Methods

This article is a targeted narrative review. To identify evidence on pharmacogenomic determinants of drug response, dosing, and toxicity in older adults, a structured literature search was conducted in PubMed/MEDLINE. The final search was performed on 23 August 2026 using the following Boolean string: (“pharmacogenomics” OR “pharmacogenetics” OR “PGx” OR “CYP2D6” OR “CYP2C19” OR “CYP2C9” OR “SLCO1B1”) AND (“elderly” OR “geriatric” OR “older adults” OR “aging”) AND (“dosing” OR “toxicity” OR “adverse drug reaction” OR “personalized medicine”).

The search was restricted to English-language, peer-reviewed publications. To focus on contemporary evidence, results were limited to studies published from 2015 to 2026. The PubMed filter “Aged: 65+ years” was applied to ensure geriatric relevance. Priority was given to systematic reviews, meta-analyses, clinical guidelines, randomized controlled trials, and prospective observational studies; case reports, non-geriatric studies, and animal studies were excluded. Where studies were conducted in general adult populations (e.g., Chen [59], Tanabe [79]), this is explicitly noted in the text as an extrapolation rather than geriatric-specific evidence. After applying date and age filters and removing duplicates, 310 records were screened by title and abstract. In addition, foundational papers published before 2015 (such as those describing classical pharmacokinetic principles) were identified through reference lists and manual screening and included when relevant. Following full-text assessment, 55 publications were considered sufficiently relevant and methodologically appropriate for inclusion in the final narrative synthesis.

To supplement the PubMed search and ensure broader coverage of the topic, a complementary search was performed in Google Scholar using similar keyword combinations (e.g., “pharmacogenetics older adults”, “CYP2D6 elderly dosing”). This step was particularly useful for identifying studies not indexed in PubMed, including some conference proceedings, preprints, and gray literature relevant to geriatric pharmacogenomics.

Reference lists of eligible primary studies and major clinical guidelines (CPIC, DPWG, Beers Criteria, STOPP/START) were manually screened to identify additional relevant reports.

The combined search strategies (PubMed and Google Scholar) identified a substantial body of literature. Because this is a narrative review, it was not preregistered, and no formal meta-analysis or risk-of-bias instrument was applied. The final reference list represents a curated selection of high-quality evidence, with emphasis on clinically actionable findings and well-validated pharmacogenetic associations in geriatric populations.

For claim-level citation, primary human studies were preferred when available, and references were assigned at the sentence level to ensure precise attribution of each claim.

Evidence was classified operationally using a four-tier system:

- Level 1: Established clinical pharmacology principles—reproducible human data on age-related Pharmacokinetic/Pharmacodynamic changes, validated across multiple cohorts or incorporated into major clinical guidelines (e.g., CPIC, DPWG, Beers, STOPP/START).
- Level 2: Supported human evidence—designed observational studies or pharmacokinetic trials with adequate sample sizes, showing consistent age-related effects but lacking comprehensive guideline endorsement.
- Level 3: Human observational or discovery evidence—data from smaller cohorts, retrospective analyses, or pharmacogenomic association studies without independent validation.
- Level 4: Preclinical or mechanistic evidence—data from animal models, in vitro systems, or single-setting human studies that require confirmation in geriatric patient populations.

These levels describe evidential maturity, not biological or clinical importance.

A second and orthogonal classification is applied to pharmacogenomic claims, because the maturity of the evidence and the kind of claim being made are independent of one another. Four levels are distinguished and are used consistently in Section 4, Section 5 and Section 11. Biological plausibility is a mechanistic argument that a variant, or an age-related change, should alter drug handling, with no human exposure data attached; on its own it never supports a prescribing recommendation. Pharmacokinetic evidence is a measured or modelled difference in exposure, clearance or concentration between genotype or age groups, and is the level at which most of the data summarised in Table 1 and Table 2 sit; a difference in exposure is not the same as a difference in outcome. Pharmacogenetic actionability means that a body such as CPIC or the DPWG [5,62,63] has published a prescribing recommendation keyed to the genotype, which is a statement about the existence of guidance rather than about whether following it improves outcomes. Demonstrated clinical utility requires evidence that acting on the genotype changes a patient-relevant outcome—an ADR, a hospitalisation, treatment failure, or mortality—and in older adults specifically this level is reached for only a minority of the gene–drug pairs discussed here. The two classifications are reported separately: the four tiers above describe how mature the underlying study design is, whereas these four levels describe what kind of claim the evidence can support. When this review recommends an action, the level on which the recommendation rests is stated; when a claim sits at a lower level than its clinical framing might suggest, this is made explicit rather than left for the reader to infer.

The four-tier system (Level 1–4) describes the maturity of the underlying study design. Separately, in Table 3, the ‘Strength of support’ column uses a different but related scheme (Direct/Indirect/Expert opinion), which describes the strength of the specific recommendation rather than the maturity of the study design. These two schemes are complementary and should not be conflated.

## 14. Conclusions

The aging process induces significant structural and functional changes across all organ systems, resulting in a reduced capacity to maintain homeostasis, particularly under physiological stress. While some organ systems may retain baseline functionality at rest, the decline in functional reserve enhances vulnerability to stressors. Key pharmacokinetic changes include alterations in body composition, such as increased adiposity and reduced total body water, which expand the volume of distribution for lipid-soluble drugs and reduce the clearance of both lipid- and water-soluble drugs. These changes collectively prolong the plasma elimination half-life of many therapeutic agents. Concurrently, aging-associated pharmacodynamic shifts, such as altered receptor sensitivity (heightened in some systems, reduced in others) and diminished compensatory homeostatic responses, further increase drug sensitivity.

The conclusion supported by the present review is accordingly a graded one, expressed at the four levels used throughout this manuscript. The biological plausibility of age-modified pharmacogenetic effects is high for the pathways reviewed. Pharmacokinetic evidence of age-related changes in exposure is strong for CYP3A (measured data), moderate for CYP2C19 (modelled data), weak for CYP2D6 (where intrinsic activity is largely preserved) and no conclusive evidence for OATP1B1. Pharmacogenetic actionability is established by CPIC and DPWG guidance for *CYP2D6*, *CYP2C19*, *CYP2C9*, *SLCO1B1*, *VKORC1*, and HLA-B*58:01, but this guidance is not age-stratified. Strong associations with an actionable screening rationale exist for HLA-B*58:01/allopurinol (case-control evidence) and *CYP2C9*/*VKORC1*/warfarin (genotype-guided trials), although prospective outcome trials in older adults remain limited. For most other gene–drug pairs, the case for testing in older adults rests on mechanistic plausibility, high exposure prevalence, and the severity of the downstream event—not on outcome evidence from geriatric cohorts. Genotype-guided prescribing in older adults is therefore mechanistically well founded and operationally actionable but not yet outcome-proven for most pairs, and this distinction should inform both clinical implementation and future research priorities.

Deprescribing using established frameworks remains the cornerstone of geriatric pharmacotherapy, reducing unnecessary medications and preventing prescribing cascades. Successful implementation of these strategies requires multidisciplinary collaboration among physicians, pharmacists, clinical pharmacologists, and other healthcare providers.

As the global population ages, the need for evidence-based, individualized pharmacotherapy in older adults will only grow. Continued research into the pharmacogenomic, pharmacodynamic, and pharmacokinetic determinants of drug response in geriatric populations—particularly in the oldest-old (≥80 years), those with frailty, and those with multiple comorbidities—is essential to ensure safer, more effective, and more equitable pharmacotherapy for older adults worldwide.

## Figures and Tables

**Table 4:** Overlap between potentially inappropriate prescribing and pharmacogenetic actionability.

Drug or Class	Gene(s)	Basis for Caution in Older Adults	Interaction Between the Two Considerations
Antidepressants with strong anticholinergic activity (amitriptyline, clomipramine, desipramine, doxepin > 6 mg/day, imipramine, nortriptyline, paroxetine)	*CYP2D6*, *CYP2C19*	Beers 2023 Table 2: Avoid—highly anticholinergic, sedating, cause orthostatic hypotension (quality of evidence high, recommendation strong). Tertiary tricyclics also appear under syncope, and antidepressants as a class under history of falls or fractures, in Beers 2023 Table 3 [53]	A poor-metabolizer genotype raises exposure to drugs already flagged for avoidance on pharmacodynamic grounds. Genotype does not make the drug appropriate; it identifies the patients in whom harm is most likely if it is used. Note that paroxetine is on this list and is itself a strong CYP2D6 inhibitor
Proton-pump inhibitors (omeprazole, pantoprazole, lansoprazole, esomeprazole, rabeprazole, dexlansoprazole)	*CYP2C19*	Beers 2023 Table 2: Avoid scheduled use beyond 8 weeks unless a high-risk indication applies—risk of *C. difficile* infection, pneumonia, gastrointestinal malignancy, bone loss and fracture [53]	Used by 16.6% of ASPREE participants; 7.9% of all participants used PPIs while carrying an actionable *CYP2C19* genotype [7]; pantoprazole and omeprazole are among the most prescribed actionable drugs in cohorts aged 65 and over. The duration limit and the genotype-based dose adjustment are independent constraints
Non-COX-2-selective NSAIDs, oral (ibuprofen, meloxicam, piroxicam, flurbiprofen, naproxen, diclofenac and others)	*CYP2C9*	Beers 2023 Table 2: Avoid chronic use unless alternatives are ineffective and a gastroprotective agent can be taken; Table 3: avoid in heart failure and in a history of gastric or duodenal ulcer; Table 6: avoid at CrCl below 30 mL/min. Celecoxib is COX-2-selective and appears in the heart-failure and kidney-function criteria rather than in the Table 2 non-selective list [53]	Third most-used cautionary class in ASPREE; 14.2% of users carried an actionable CYP2C9 genotype [7]. Reduced CYP2C9 function prolongs exposure in a class already flagged for avoidance, and the renal criterion applies concurrently
Warfarin	*CYP2C9*, *VKORC1*, *CYP4F2*	Beers 2023 Table 2 (new in the 2023 update): avoid starting warfarin as initial therapy for non-valvular atrial fibrillation or venous thromboembolism unless DOACs are contraindicated or there are substantial barriers to their use (quality high, recommendation strong); continuation may be reasonable in long-term users with well-controlled INR. Table 5: Avoid combining warfarin with amiodarone, ciprofloxacin, macrolides other than azithromycin, trimethoprim-sulfamethoxazole or SSRIs [53]	Because the 2023 criteria discourage initiating warfarin in this age group, the population in which *CYP2C9* and *VKORC1* genotyping alters an initial dose is narrower than under earlier criteria. The listed interactions are also phenoconversion mechanisms
Sulfonylureas (glimepiride, glyburide/glibenclamide, glipizide, gliclazide)	*CYP2C9*	Beers 2023 Table 2: Avoid as first- or second-line monotherapy or add-on therapy; if a sulfonylurea is used, prefer a short-acting agent (glipizide) over a long-acting one (glyburide, glimepiride), which confer a higher risk of prolonged hypoglycaemia [53]	The 2023 update expanded this criterion from long-acting agents only to the whole class. Chlorpropamide was removed because it is no longer marketed in the United States [53]
Tramadol	*CYP2D6*	Beers 2023 Table 4 (use with caution): May exacerbate or cause SIADH or hyponatraemia, with sodium monitoring advised on initiation or dose change; Table 6: reduce the immediate-release dose and avoid the extended-release form at CrCl below 30 mL/min. Opioids as a class appear under delirium and under history of falls or fractures in Table 3 [53]	Poor metabolizers obtain reduced analgesia from bioactivation while retaining the non-opioid adverse effects; ultrarapid metabolizers are exposed to excess active metabolite. Neither is addressed by the criteria themselves
Codeine	*CYP2D6*	Not named individually in the Beers 2023 criteria; opioids as a class appear under delirium and under history of falls or fractures in Table 3, and under the opioid–benzodiazepine and opioid–gabapentinoid interactions in Table 5 [53]	Same bioactivation logic as tramadol. A co-prescribed CYP2D6 inhibitor converts a normal metabolizer into a functional poor metabolizer, which the explicit criteria do not capture
SSRIs (citalopram, escitalopram, sertraline)	*CYP2C19*	Beers 2023 Table 4 (use with caution): SSRIs may exacerbate or cause SIADH or hyponatraemia; Table 3: antidepressants under history of falls or fractures; Table 5: avoid combining with warfarin. The maximum daily citalopram dose above age 60 is a regulatory label restriction and not a Beers criterion [53]	The age-based label ceiling and the genotype-based dose reduction are separate constraints that apply concurrently rather than substituting for one another
Clopidogrel	*CYP2C19*	Not a potentially inappropriate medication in the Beers 2023 criteria. The criteria instead position clopidogrel as the safer comparator: prasugrel and ticagrelor are placed in Table 4 for caution in adults aged 75 and over because both increase major bleeding relative to clopidogrel [53]	The most frequently used actionable drug in the cohorts aged 65 and over and in Danish nursing homes [69]. Its pharmacogenetic relevance stands on its own and should not be presented as reinforcing a potentially inappropriate flag
Statins (simvastatin, atorvastatin and others)	*SLCO1B1*, *ABCG2*, *CYP2C9*	Not included in the Beers 2023 criteria [53]	The most-used cautionary class in ASPREE at 29.3% [7]. Volume of use rather than per-patient risk is what makes this the highest-yield gene–drug pair in this age group [63]

## Data Availability

No new data were created or analyzed in this study. Data sharing is not applicable to this article.

## Abbreviations

The following abbreviations are used in this manuscript:

ADME	Absorption, Distribution, Metabolism, and Excretion
ADR	Adverse Drug Reaction
ANOVA	Analysis of Variance
AUC	Area Under the Curve
CI	Confidence Interval
CKD	Chronic Kidney Disease
CPIC	Clinical Pharmacogenetics Implementation Consortium
CYP	Cytochrome P450
DDGI	Drug–Drug–Gene Interaction
DDI	Drug–Drug Interaction
DOAC	Direct Oral Anticoagulant
DPWG	Dutch Pharmacogenetics Working Group
eGFR	Estimated glomerular filtration rate
HIV	Human Immunodeficiency Virus
HLA	Human Leukocyte Antigen
INR	International Normalized Ratio
NSAID	Non-Steroidal Anti-Inflammatory Drug
OATP	Organic Anion Transporting Polypeptide
PACE	Program of All-Inclusive Care for the Elderly
PBPK	Physiologically Based Pharmacokinetic
PET	Positron Emission Tomography
P-gp	P-glycoprotein
PGx	Pharmacogenomics/Pharmacogenetics
PPI	Proton Pump Inhibitor
SCAR	Severe Cutaneous Adverse Reaction
SNRI	Serotonin–Norepinephrine Reuptake Inhibitor
SSRI	Selective Serotonin Reuptake Inhibitor
START	Screening Tool to Alert to Right Treatment
STOPP	Screening Tool of Older Persons’ Prescriptions
TTR	Time in Therapeutic Range
UGT	Uridine Diphosphate Glucuronosyltransferase
